# A metalloprotease produced by larval *Schistosoma mansoni* facilitates infection establishment and maintenance in the snail host by interfering with immune cell function

**DOI:** 10.1371/journal.ppat.1007393

**Published:** 2018-10-29

**Authors:** Jacob R. Hambrook, Alèthe L. Kaboré, Emmanuel A. Pila, Patrick C. Hanington

**Affiliations:** School of Public Health, University of Alberta, Edmonton, Alberta, Canada; Case Western Reserve University, UNITED STATES

## Abstract

Metalloproteases (MPs) have demonstrated roles in immune modulation. In some cases, these enzymes are produced by parasites to influence host immune responses such that parasite infection is facilitated. One of the best examples of parasite-mediated immune modulation is the matrix metalloprotease (MMP) leishmanolysin (Gp63), which is produced by species of the genus *Leishmania* to evade killing by host macrophages. Leishmanolysin-like proteins appear to be quite common in many invertebrates, however our understanding of the functions of these non-leishmania enzymes is limited. Numerous proteomic and transcriptomic screens of schistosomes, at all life cycle stages of the parasite, have identified leishmanolysin-like MPs as being present in abundance; with the highest levels being found during the intramolluscan larval stages and being produced by cercaria. This study aims to functionally characterize a *Schistosoma mansoni* variant of leishmanolysin that most resembles the enzyme produced by *Leishmania*, termed SmLeish. We demonstrate that SmLeish is an important component of *S*. *mansoni* excretory/secretory (ES) products and is produced by the sporocyst during infection. The presence of SmLeish interferes with the migration of *Biomphalaria glabrata* haemocytes, and causes them to present a phenotype that is less capable of sporocyst encapsulation. Knockdown of SmLeish in *S*. *mansoni* miracidia prior to exposure to susceptible *B*. *glabrata* reduces miracidia penetration success, causes a delay in reaching patent infection, and lowers cercaria output from infected snails.

## Author summary

Parasitic flatworms, or digenetic trematodes, cause a wide range of diseases of both medical and agricultural importance. Nearly all species of digenea require specific species of snail for their larval development and transmission. The factors underpinning snail host specificity and how they dictate infection establishment and maintenance are interesting areas of research, both from the perspective of evolutionary immunology and potential application in the design of tools that aim to prevent trematode transmission. Currently, our understanding of snail-trematode associations is one-sided, being predominantly derived from studies that have focused on the snail immune response, with almost nothing known about how the parasite facilitates infection. Metalloproteases, such as leishmanolysin, are proteolytic enzymes; some of which are produced by parasites to influence host immune responses and facilitate parasite success upon encountering the host defense response. Here, we have functionally characterized a leishmanolysin-like metalloprotease (SmLeish) from *Schistosoma mansoni*, a causative agent of human schistosomiasis, which afflicts over 260 million people globally. We demonstrate that SmLeish is associated with developing sporocysts and is also located in *S*. *mansoni* excretory/secretory products and interferes with snail haemocyte morphology and migration. Knockdown of SmLeish in *S*. *mansoni* miracidia prior to exposure to *Biomphalaria glabrata* snails reduces miracidia penetration success, delays attainment of patent infections, and lowers cercaria output from infected snails.

## Introduction

Compatibility between parasites and their hosts is influenced by a wide variety of immune and immunosuppressive factors that arise over the co-evolutionary history of a host/parasite relationship. This is evident when examining the parasitism of gastropod molluscs by digenean trematodes. Snails rely heavily upon an immune response comprised of soluble immune effector molecules and immune cells, termed haemocytes, that serve to encapsulate and kill sporocysts [1]. Successful trematodes often dampen and/or completely negate specific functions associated with haemocytes, such as the capacity to encapsulate and kill the invading parasite, while also conferring a level of protection to other invading trematodes that would typically be targeted and killed [2–4]. This process is mediated by the release of products that impact haemocyte mobility, phagocytic activity, attachment, spreading, and production of reactive oxygen species (ROS) [5–7].

The specific factors excreted/secreted by a trematode (termed ES products) that modulate the snail immune response to facilitate parasite infection establishment and persistence are not well known. Based on phenotypic observations of haemocyte rounding and an inability to migrate towards a trematode sporocyst when impacted by ES products of specific trematodes [8], factors that impact the extracellular matrix, such as matrix metalloproteases, might be responsible. Proteomic and transcriptomic screens of schistosomes at all life cycle stages have identified predicted proteins with the hallmark identifiers of metalloproteases, and many are produced in abundance throughout the *S*. *mansoni* life cycle [9–15]; with the highest levels being found during the intramolluscan larval stages and in cercaria [13–15].

Metalloproteases are part of a larger group of proteolytic enzymes that also encompasses aspartic, glutamic, serine, cysteine, and threonine proteases. They can either be endopeptidases or exopeptidases [16]. The use of a zinc metal ion to perform hydrolysis reactions is the defining characteristic of metalloproteases. In most cases, these enzymes possess a conserved zinc-binding motif (HExxH) in which the two histidines coordinate the zinc ion and the glutamate act as a general base in the catalytic reaction. Metalloproteases can be categorized according to their catalytic mechanism, their substrates and products, or their structural homology [16]. A wide variety of physiological processes such as morphogenesis, peptide and hormone processing, cell adhesion and fusion, proliferation, migration, apoptosis, angiogenesis, and inflammation are mediated by metalloproteases [16, 17].

One of the most studied metalloproteases in the context of parasitic infection is leishmanolysin, which is also referred to as Gp63. Olivier et al. (2012) [18] reported Gp63, a major leishmania surface antigen, as a zinc-dependent metalloprotease. Leishmanolysin is capable of cleaving casein, complement component C3, gelatin, albumin, haemoglobin, immunoglobulin C3 and fibrinogen [19–21]. It is localized at the surface of the plasma membranes of leishmania, but also can be excreted and released into the host [18]. Gp63 participates in immunomodulatory activities that facilitate infection of host macrophages and protection of leishmania within the macrophage from degradation in the phagolysosome. It is known to assist in the avoidance of complement-mediated lysis of leishmania through the cleavage of C3b [22]. The product of this cleavage, iC3b triggers macrophages via the Mac-1 complement receptor, which results in increased parasite internalization, thereby facilitating infection [23]. Additionally, Gp63 is thought to interact with both complement and fibronectin receptors to facilitate leishmania entrance into macrophages [22, 24, 25]. It is also capable of degrading the extracellular matrix of host macrophages, which accelerates mobility of leishmania into these cells [26]. More specifically, the hydrolysis of Protein Kinase C (PKC) substrates such as myristoylated alanine-rich C kinase substrate (MARCKS) and MARCKS-related proteins (MRP), found in macrophages, alters the PKC signaling pathway in the infected host macrophages leading to inhibition of the production of anti-microbial agents like reactive oxygen species (ROS) [18]. Hence, via cleavage and/or degradation, leishmanolysin creates a favorable milieu in the host that facilitates parasite survival by altering the host immune response through signaling pathway inhibition at various stages of infection.

Leishmanolysin can also be termed invadolysin in species other than leishmania, and has been identified in bacteria, plants, and invertebrate and vertebrate animals [27]. These sets of homologous proteins are all generally classified in the M8 family of metzincin metalloproteases [27]. They are usually endopeptidases without exopeptidase activity [19]. These proteins appear to be commonly associated with host immunosuppression by trypanosomatid parasites. For example, Gp63 homologues have also been implicated in the immune evasion strategies of species of the genus *Trypanosoma*. One of the known evasive and protective functions of *T*. *brucei* Gp63 includes the removal of variable surface glycoproteins on the surface of the bloodstream stages of the parasite to evade host recognition and killing [28]. *T*. *cruzi* trypomastigotes entry into red blood cells is inhibited in neutralization assays using antibodies raised against Gp63 [29, 30]. *T*. *carassii* Gp63 interacts with macrophages of the goldfish host by inhibiting ROS and nitric oxide production, alteration of the phospho-tyrosine protein patterns resulting in downregulation of pathogen and cytokine-induced inflammatory responses of monocytes and macrophages [31]. This family of proteins has yet to be functionally characterized in the context of helminth infection.

Numerous analyses of the transcriptome and proteome of *S*. *mansoni* during various life cycle stages has implicated leishmanolysin-like factors in the establishment and maintenance of *S*. *mansoni* infection of the snail host. In this study, we have functionally characterized an *S*. *mansoni* leishmanolysin, showing that it suppresses the snail host immune response during the early stages of the intramolluscan infection. We demonstrate that this *S*. *mansoni* leishmanolysin (ID: CCD79314.1), termed SmLeish for the purposes of this manuscript, is an important component of *S*. *mansoni* ES products and is also present on the sporocyst surface. Additionally, we demonstrate that SmLeish facilitates the infection of *Biomphalaria glabrata* snails through interference with *B*. *glabrata* haemocyte migration, causing them to be less capable of sporocyst encapsulation. Knockdown of SmLeish in *S*. *mansoni* miracidia prior to exposure to *B*. *glabrata* significantly influences the kinetics of the infection, reducing miracidia penetration success, the proportion of snails that shed cercaria and the number of shed cercaria per infected snail.

## Results

### Abundance of the SmLeish transcript and soluble protein is highest during the early stages of the intramolluscan infection and is associated with the developing sporocyst

**Fig 1:**
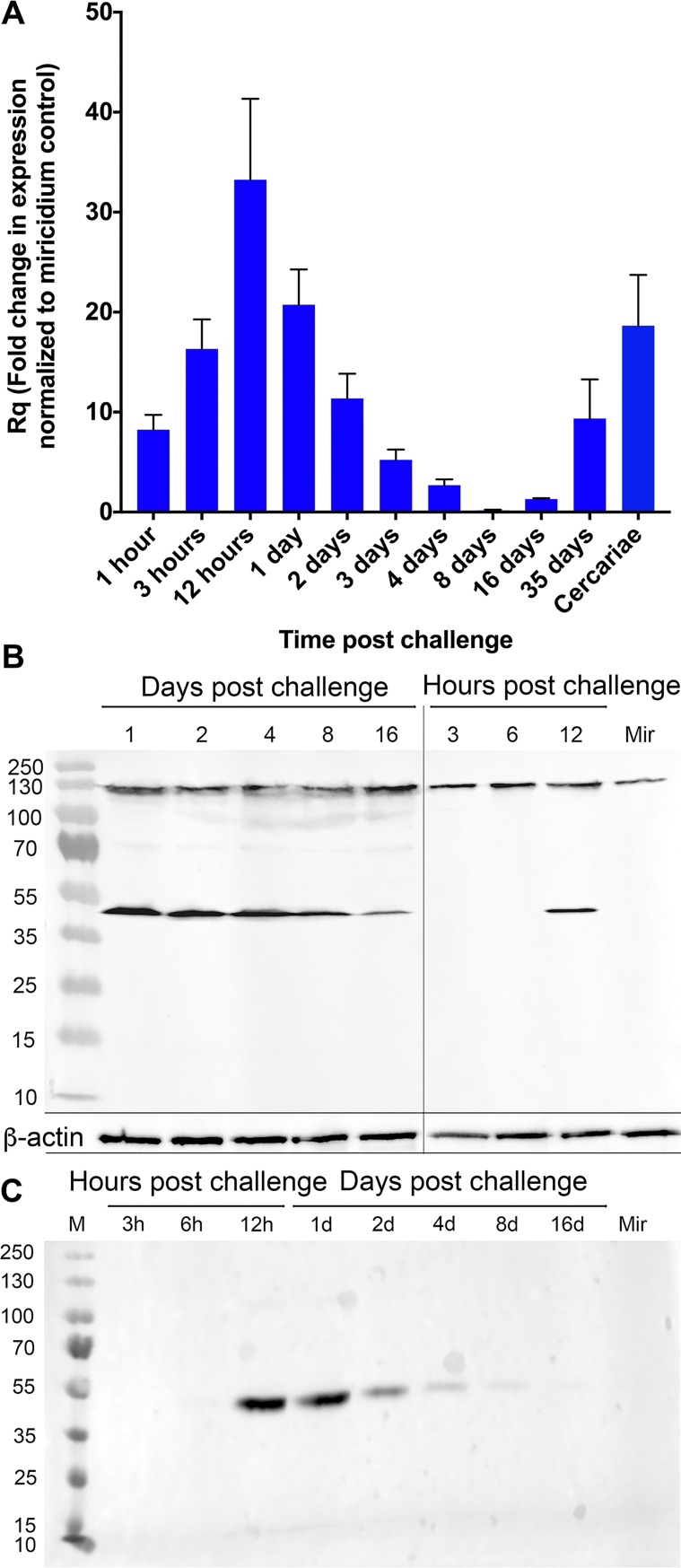
Assessment of SmLeish expression during *S*. *mansoni* intramolluscan infection. (A) Quantitative RT-PCR analysis of SmLeish transcript abundance during critical points of *S*. *mansoni* intramolluscan development. M-line snails (*n* = 5 for each time point) were challenged with 5 miracidia of PR-1 *S*. *mansoni*. Fold change of SmLeish transcription normalized to a miracidium control show an increase in transcription over the initial 12 hours post infection. As infection progresses, transcript abundance relative to control declines until day 35 post challenge, a point at which cercaria begin to emerge from the infected snails. Cercaria emergence correlates with a renewed spike in SmLeish transcription. (B) Western blot analysis of whole-snail lysates confirms the qRT-PCR data, demonstrating that the increased expression is likely due to the emergence and relative increase in the abundance of a ~48kDa protein and not the larger full-length SmLeish. ß-actin is used as a loading control. (C) Western blot analysis of *B*. *glabrata* plasma demonstrates that the ~48kDa protein likely represents a soluble SmLeish that appears around 12 hours post challenge.

**Fig 2:**
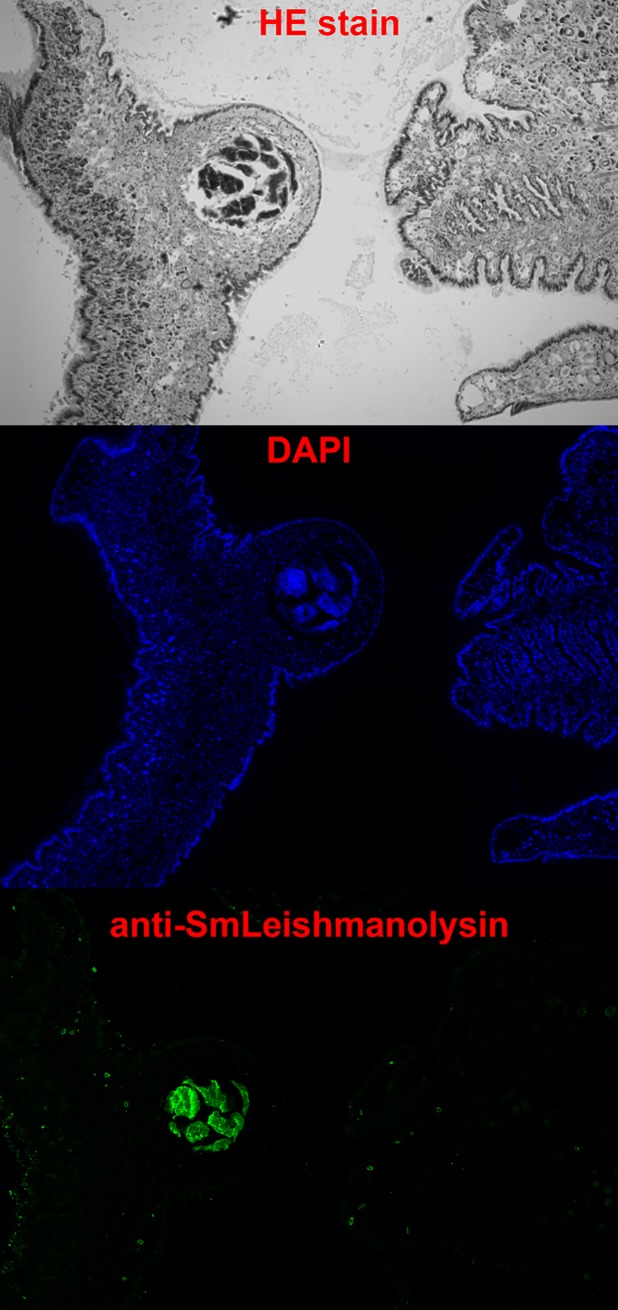
Confirmation that SmLeish is associated with the larval *S*. *mansoni* sporocyst within the intramolluscan environment. The anti-SmLeish antibody was used to probe histological sections of *S*. *mansoni*-challenged *B*. *glabrata* to visualize the context of SmLeish expression within the snail. At all time points observed, SmLeish expression was confined to the sporocyst. Depicted is a day 16 sporocyst within the head-foot of an M-line *B*. *glabrata*.

SmLeish is transcribed at all stages of the intramolluscan development of *S*. *mansoni*. Relative transcript abundance in comparison to the endogenous control, *S*. *mansoni* GAPDH, which allows for confirmation of *S*. *mansoni* presence and semi-quantification of *S*. *mansoni* within *B*. *glabrata* tissues, peaks at 12-hours post challenge (hpc) with a magnitude 33.2-fold higher than pre-challenge miracidium. SmLeish transcript abundance declines from 12-hpc until cercaria emergence begins around 35 days post challenge (dpc), at which time it rises again to 9.4-fold higher than pre-challenge miracidium. A 18.6-fold higher abundance of SmLeish transcript in isolated shed cercariae suggests that this later increase in SmLeish transcription at 35 dpc is associated with cercariae generation (Fig 1A). Quantitative RT-PCR assessment of SmLeish transcript abundance was confirmed by Western blot analysis of challenged whole-snail lysates. Probing with the anti-SmLeish antibody reveals a ~130kDa protein that is very close in size to the estimated 125.9kDa of the complete SmLeish protein. This protein displays relatively stable abundance during infection up to 16-dpc. The constitutive expression of this larger protein is contrasted by dynamic expression of a smaller ~48kDa protein that appears at 12 hpc and persists up to 16 dpc (Fig 1B). The appearance of this smaller protein in challenged whole-snail lysates correlates with a ~48kDa protein that is detected by the anti-SmLeish antibody in *S*. *mansoni-*infected M-line *B*. *glabrata* plasma, suggesting that this smaller protein is a soluble version of SmLeish (Fig 1C). Investigation using two alternative RT-qPCR assays that targeted the 5’ and 3’ ends of the SmLeish transcript showed little variance in SmLeish transcript abundance when compared to the primary primer and probe set used, suggesting that alternative splicing of the SmLeish transcript is not responsible for the appearance of the ~48kDa protein (Table 1, S1 Fig). Immunofluorescent detection of *S*. *mansoni* sporocysts in histological sections of infected *B*. *glabrata* using the anti-SmLeish polyclonal antibody suggests that SmLeish is produced in association with the larval parasite and would likely be in close proximity to surrounding haemocytes (Fig 2).

**Table 1:** Primers and probe used in this study.

Primer (Position)	Sequence 5’→3’	
SmLeish FWD Set 1 (2875–2895)	CCTCATCGCTTACCAGAATGT	
SmLeish Probe Set 1 (2897–2920)	TTACAAATCCACCCACCCACTCTGG
SmLeish REV Set 1 (2948–2969)	TGGTTAGTATGCGCTCGAATTA
SmLeish FWD Set 2 (749–770)	GTGATCTGAGGCACTATCTTCG
SmLeish Probe Set 2 (790–817)	CAGTACTTTGAAGACCCGAGACTTGGTT
SmLeish REV Set 2 (845–865)	CGGTAAATGGCACATAAGCTG
SmLeish FWD Set 3 (1503–1525)	ATCCTATGCTTTCCTACGTGAAG
SmLeish Probe Set 3 (1536–1561)	ACCACGTACACCTAGAGACCCTCAAA
SmLeish REV Set 3 (1633–1652)	GCAGAAACCCAGATTCGTTG
SmLeish expression *Fwd	*CAC C***AG GAG G**AG GAG AGC CAT GGT ACC CTG TTC AAG
SmLeish expression Rev	TTG TTT AAT TGA TCT ACG CCT G

*Forward primer modified by adding CACC nucleotides (italicized) and ribosome binding sequence (bold font) for efficient cloning and expression.

### Recombinant SmLeish possesses MMP functionality

**Fig 3:**
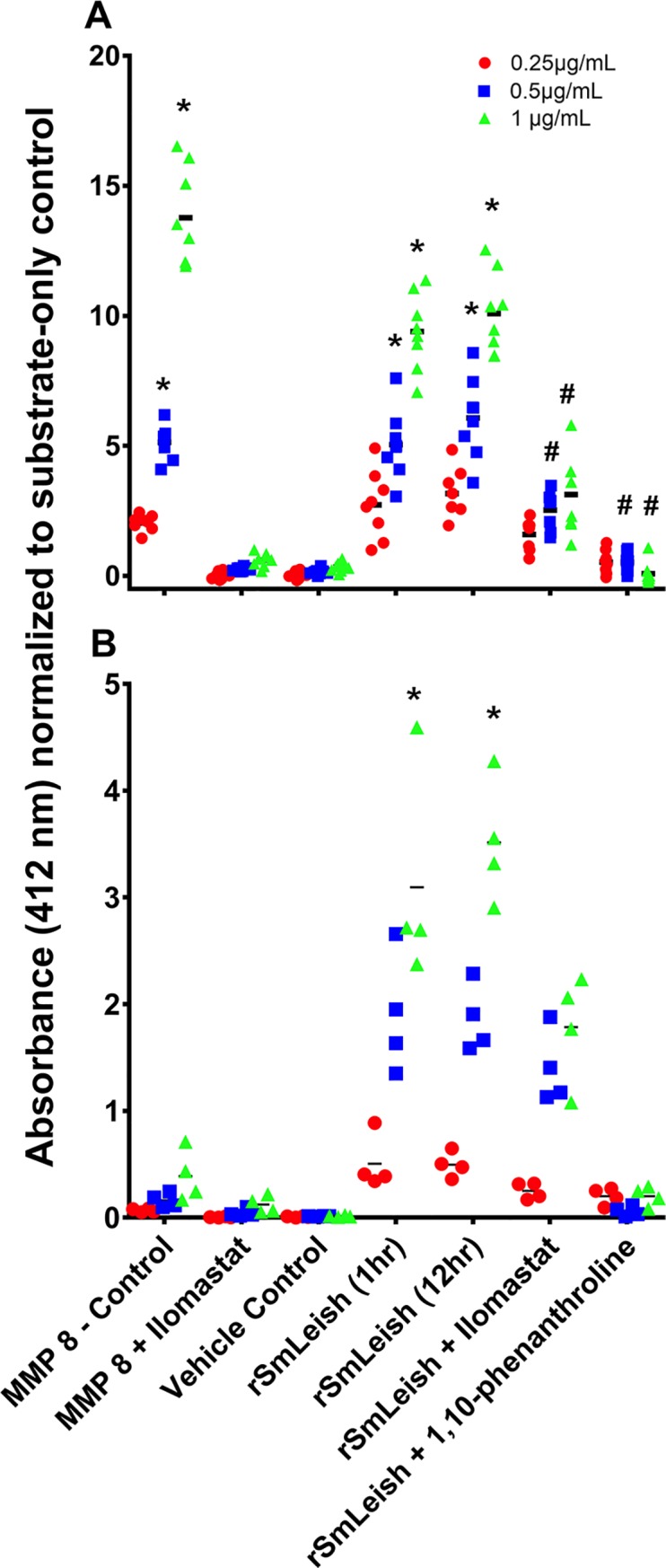
Recombinant SmLeish is a functional matrixmetalloprotease. (A) Metalloprotease activity of activated (trypsinized) rSmLeish prior to incubation with the MMP8 substrate. Recombinant SmLeish is able to cleave human MMP8 substrate in a dose-dependent fashion, but is only partially inhibited by Ilomastat, and almost completely inhibited by 1,10-phenanthroline. (B) Unlike human MMP8, which requires trypsin activation in order to display functional activity, rSmLeish does not require prior trypsinization in order to display MMP activity. However, rSmLeish enzymatic activity is significantly reduced if trypsin activation does not occur prior to assessment. Treatment of rSmLeish with 5μg/mL trypsin for 12 hours did not significantly alter the MMP activity compared to the 1 hour at 10μg/mL. In both experiments, three initial concentrations (0.25 μg/mL 0.5 μg/mL and 1μg/mL) of the enzymes were used. Enzymatic activity indicates the concentration of the product (sulfhydryl group) formed, measured via OD_412_ absorbance values after 2-hours incubation with the substrate. Significant differences between treatments and the vehicle control are signified by *, significant differences between inhibitor treatments and rSmLeish are signified by #.

SmLeish is predicted to possess two peptidase M8 superfamily domains, the first between amino acids 20–405, and the second between 429 and 737, that are characteristic of leishmanolysin proteins (S2 Fig). A clear signal peptide is present in the first 20 amino acids, however no traditional transmembrane region is predicted. SmLeish shares the highest amino acid identity with other leishmanolysin-like metalloproteases, however, with respect to the well-characterized human matrix metalloproteases, SmLeish is most similar to MMP8 (14.1% amino acid identity). To confirm that SmLeish functions as a metalloprotease, it was compared to human MMP8 in an MMP8 activity ELISA. The human MMP8 displayed dose-dependent and trypsin-activation dependent activity. Its activity was inhibited using the human MMP8 inhibitor (Ilomastat) control provided with the ELISA kit (Fig 3A and 3B). Recombinant SmLeish (rSmLeish) also displayed a dose-dependent activity that was partially inhibited using the Ilomastat inhibitor, and almost completely abrogated using a different MMP inhibitor, 1,10-phanthroline (Fig 3A). Trypsin activation of SmLeish was not required for MMP activity, however, activity was diminished by ~4x if trypsin was not used (Fig 3B). Prolonged incubation of rSmLeish with trypsin at a concentration of 5μg/mL for 6 or 12 hours resulted in more efficient cleavage of the pro-rSmLeish (S3B Fig), however, rSmLeish treated with trypsin for 12 hour did not significantly enhance the MMP activity compared to the 1-hour treatment (Fig 3).

To confirm that trypsin treatment cleaves rSmLeish, and to determine whether the ~48kDa protein that appears during *S*. *mansoni* infection of *B*. *glabrata* is generated by trypsin cleavage, rSmLeish was exposed to 1, 5 and 10μg/mL of trypsin for 1 hour. Five distinct bands were visualized by Western blot using the anti-SmLeish antibody, and these cleavage products became more resolved when 5 or 10μg/mL trypsin was used. The five bands were sent for tandem MS analysis and three returned conclusive results. The largest protein (~130kDa), along with two smaller proteins, one at ~50 kDa and the other at ~35kDa, all matched peptides to SmLeish. The MS analysis did not provide sufficient data to map the exact locations within SmLeish from which the smaller proteins originated, however both analyses returned only peptides from the C-terminal region of the rSmLeish protein (S3C Fig).

### Soluble SmLeish is a component of *S*. *mansoni* ES products and rSmLeish inhibits M-line *B*. *glabrata* haemocyte migration

**Fig 4:**
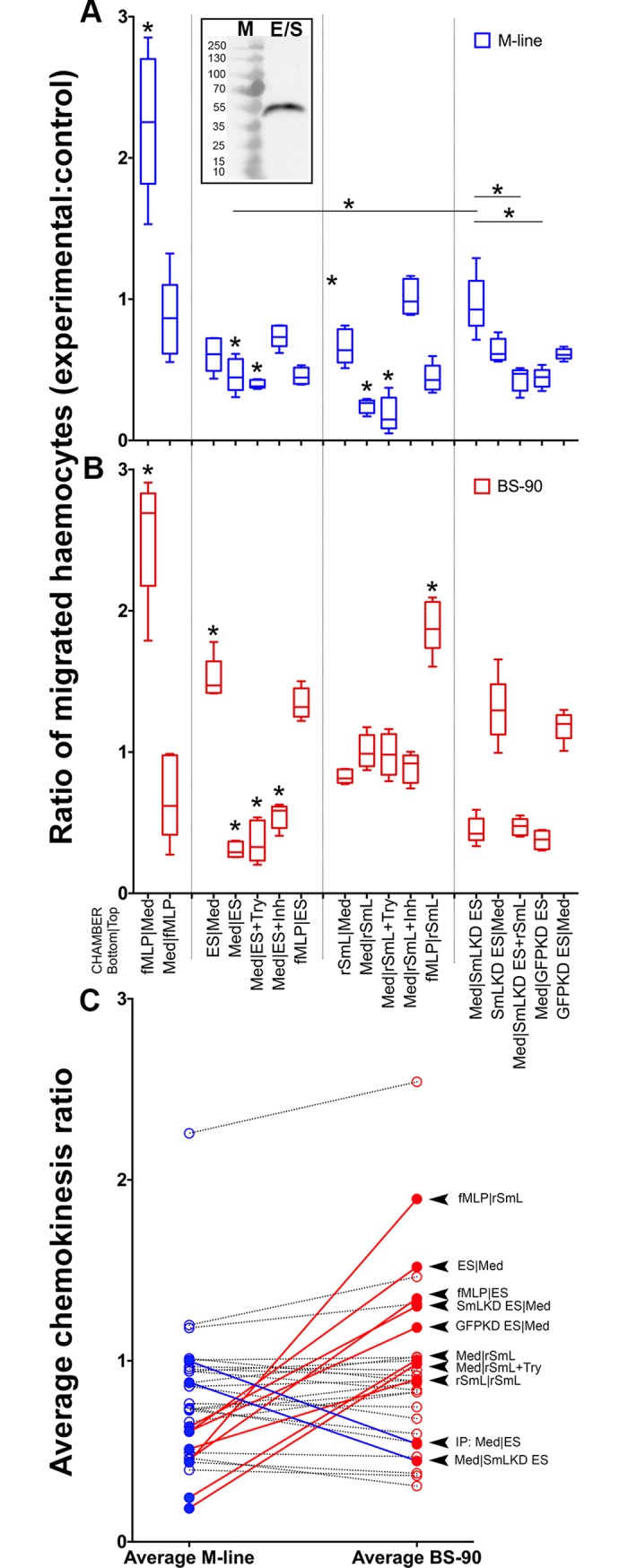
Recombinant SmLeish interferes with M-line *B*. *glabrata* haemocyte motility. Directional migration of M-line and BS-90 haemocytes across a 5μM porous membrane was assessed to determine the impact of rSmLeish on haemocyte motility. Migration is presented as a ratio comparing the number of haemocytes that have migrated to the underside of the membrane in an experimental group to a control group (medium only in both upper and lower chambers) where both haemocyte groups originate from the same snail. fMLP was used as a positive control. (A) *S*. *mansoni* excretory/secretory (ES) products (which are shown to contain SmLeish in the inset Western blot) inhibit migration of M-line haemocytes across the membrane when incubated with the haemocytes in the upper well. Recombinant SmLeish (rSmL) inhibits migration of M-line haemocytes across the membrane when incubated with the haemocytes in the upper chamber and this effect was slightly enhanced by pretreatment of rSmL with trypsin (Try). Knockdown of SmLeish prior to generation of ES products abrogated the negative impact of ES products on M-line haemocyte migration and the effect was rescued by addition of rSmLeish to the SmLeish KD ES products. (B) E/S products are attractive to BS-90 haemocytes when in the lower well. rSmLeish had no impact on BS-90 haemocytes and was not attractive to BS-90 haemocytes. Knock down of SmLeish in larval E/S products did not abrogate the attractive nature of E/S products to BS-90 haemocytes. * represents significant difference in haemocyte migration between a treatment and its corresponding control group, or to a specific control as indicated by a solid line. (C) Comparison of the mean values in each treatment between M-line and BS-90 *B*. *glabrata* haemocytes identified 10 treatments that yielded significantly different results. A value above 1 indicates migration across the membrane greater than control, a value less than 1 indicates less migration than control. Each treatment is connected between the M-line and BS-90 datasets to aid in visualizing the differences between the different snail haemocytes. A red line indicates that the treatment identified on the left induced more migration in BS-90 haemocytes than M-line, a blue line indicates more migration in M-line haemocytes than BS-90.

*S*. *mansoni* ES products are known to negatively impact haemocyte recruitment to the sporocyst and often lead to a phenotypic rounding and loss of haemocyte adherence *in vitro*. SmLeish was confirmed to be an important component of ES products (Fig 4 inset), and to test whether SmLeish contributes to alterations of haemocyte function, its impact on chemokinetic activity of haemocytes from M-line and BS-90 *B*. *glabrata* was assessed. Haemocytes isolated from individual snails were equally separated into two pools and placed in the top chamber of a cell migration apparatus and then one pool was treated and the other served as a control. Treatments were placed in the bottom well to stimulate migration across the membrane and assess chemoattraction, the upper chamber to determine whether contact exposure influenced cell movement to the membrane underside, or both chambers to assess chemokinesis. The ratio of migrated (underside of membrane) haemocytes in the control haemocyte pools, which was exposed to medium in both the upper and lower chambers, was compared to the experimental group of haemocytes from each snail and a ratio of experimental migration to control migration was established, with values >1 reflecting more migration in the experimental group and values <1 reflecting less.

The positive control, fMLP, induced haemocyte migration at a ratio of 2.25±0.49:1 in M-line snails and 2.53±0.43:1 in BS-90 snails. Adding fMLP to the top chamber abrogated directional migration of the haemocytes to the membrane underside (Fig 4A), as did having fMLP in both chambers (S4 Fig).

Placing either ES products or rSmLeish in the upper chamber with M-line haemocytes significantly reduced the number of haemocytes that crossed the membrane compared to controls (0.44±0.07:1 and 0.24±0.05:1 respectively) and treating rSmLeish with trypsin prior (which had no effect on its own following deactivation [S2 Fig]) to haemocyte exposure in the upper chamber slightly enhanced the observed reduction in migration (0.19±0.12:1). Having ES products or rSmLeish in the bottom chamber did not significantly impact M-line haemocyte migration compared to controls (0.61±0.12:1 and 0.66±0.12:1 respectively), although there was a trend towards reducing haemocyte migration across the membrane (Fig 4A). Removal of SmLeish from ES products or rSmLeish by immunoprecipitation prior to incubation significantly abrogated the negative effect on M-line haemocyte migration (0.96±0.13:1) (S4 Fig), as did co-treating the haemocytes in the top well with rSmLeish and 1,10-phenanthroline (1.01±0.13:1) (Fig 4A).

The impact of ES products and rSmLeish on reducing haemocyte migration were not observed when BS-90 *B*. *glabrata* haemocytes were assessed. In fact, ES products, but not rSmLeish, were significantly attractive to BS-90 haemocytes when placed in the bottom well (1.51±0.15:1 and 0.82±0.05:1 respectively), suggesting that the impact of SmLeish (or lack of impact) on haemocytes of BS-90 *B*. *glabrata* may represent one of the reasons that this strain of *B*. *glabrata* is refractory to infection by many strains of *S*. *mansoni*. Removal of SmLeish from ES products by immunoprecipitation did not significantly eliminate the attractive properties (1.46±0.33:1) (S4 Fig), nor did pretreatment of rSmLeish with trypsin (0.98±0.15:1) or co-treatment with 1,10-phenanthroline (0.89±0.10:1) (Fig 4A).

To confirm that the loss of chemokinetic activity in M-line haemocytes incubated with *S*. *mansoni* ES products was in fact due to SmLeish, ES products from SmLeish knockdown parasites were tested. Knockdown of SmLeish significantly abrogated the inhibition of chemokinesis observed in M-line haemocytes (0.96±0.21:1) compared to both the normal ES products (0.46±0.12:1) and GFP knockdown ES product (0.44±0.07:1) controls. Moreover, the loss of inhibition could be rescued if rSmLeish was added back to the SmLeish knockdown ES products prior to incubation with M-line haemocytes in the upper chamber (0.43±0.08:1) (Fig 4A). No significant impacts of SmLeish knockdown were observed when ES products from knockdown parasites were applied to BS-90 haemocytes (Fig 4A).

Additional differences between the responses of M-line and BS-90 haemocytes emerged when fMLP was included in the bottom chamber and ES products or rSmLeish in the top chamber. While fMPL was able to induce significant migration of BS-90 haemocytes across the membrane when either ES products or rSmLeish were applied in the top chamber (1.34±0.11:1 and 1.89±0.19:1 respectively), the suppressive effects of ES products (0.46±0.06:1) and rSmLeish (0.44±0.10:1) prevented migration when M-line haemocytes were used (Fig 4A and 4B).

Further comparison between the effects of ES products and rSmLeish on M-line and BS-90 haemocytes yielded 10 treatments in which haemocyte migration significantly differed between the two *B*. *glabrata* strains. Eight of the 10 treatments (depicted bottom|top; 1. fMLP|rSmLeish, 2. ES|Medium, 3. fMLP|ES 4. SmLeish-KD ES|Medium, 5. GFP-KD ES|Medium, 6. Medium|rSmLeish, 7. Medium|rSmLeish+Try and 8. rSmLeish|rSmLeish) all resulted in fewer M-line haemocytes migrating compared to controls (a migration ratio <1) while BS-90 haemocytes in these same treatments resulted in migration ratios near to, or above 1. The two remaining of the 10 significantly different treatments, Medium|SmLeish-immunoprecipitated ES and Medium|SmLeish KD ES resulted in the opposite trend, where M-line haemocytes in these treatment groups migrated similar to controls and BS-90 haemocytes significantly less than controls (Fig 4B).

### Knockdown of SmLeish delays *S*. *mansoni* infection establishment and cercarial output, but does not reduce long-term infection success in M-line *B*. *glabrata*

Knockdown of SmLeish using siRNA successfully reduced transcript abundance as early as 2-day post transfection *in vitro* (S5 Fig). Knockdown was statistically significant from day 3 post transfection onward compared to time-matched controls where the relative fold change in transcript abundance compared to miracidium reached 11.69±3.15 at day 4 post transfection in controls, compared to 0.94±0.33 in the knockdown group (S5 Fig). Western blot analysis indicates that the abundance of the larger ~130kDa protein declined following knockdown, however, most noticeable was the complete absence of the ~48kDa soluble SmLeish (S5 Fig).

**Fig 5:**
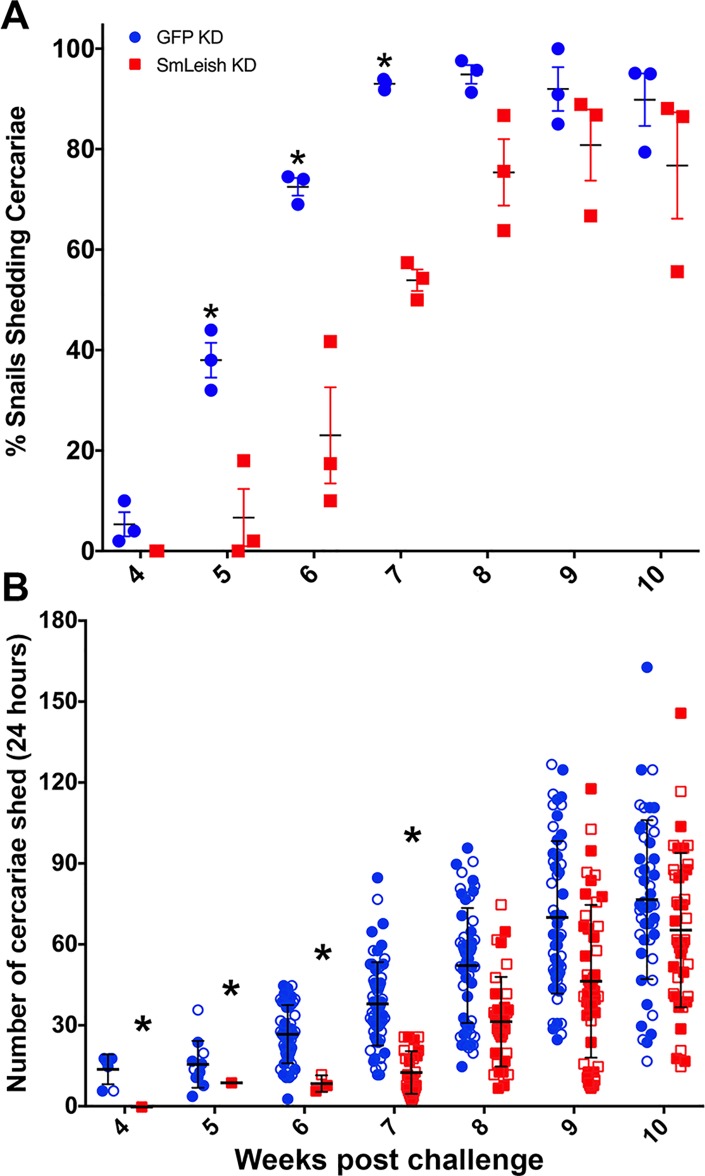
Knockdown of SmLeish influences *S*. *mansoni* infection kinetics and cercarial output. (A) Knockdown of SmLeish prior to challenge of M-line *B*. *glabrata* reduced the early infection success of the parasite as measured by the number of *S*. *mansoni*-challenged *B*. *glabrata* that shed cercaria at 4, 5, 6, 7, 8, 9 and 10 weeks post challenge compared to GFP knockdown control parasites. Long-term infection success was not statistically changed after 8-weeks post challenge, suggesting that knockdown of SmLeish may reduce the number of miracidia (out of the 5 each snail was exposed to) that successfully infected the snail. However, significant differences were observed (*) at 5, 6 and 7 weeks post challenge. Shown are the mean % snails shedding cercariae resulting from three independent trials, with mean and standard error. (B) Cercaria output was also significantly influenced by SmLeish knockdown early in the shedding phase of the infection, at weeks 4, 5, 6, and 7 post challenge (*). However, parallel to the number of snails shedding, cercaria outputs were not statistically different by week 8 post challenge. Shown are the individual cercaria output numbers per infected snail at each time point. Closed and open markers distinguish between the two trials, with mean and standard deviation shown.

Knockdown of SmLeish in *S*. *mansoni* miracidia prior to exposure to M-line *B*. *glabrata* significantly influenced the kinetics of the infection, reducing the proportion of snails that shed cercaria until 8-weeks post challenge. At 4-weeks post challenge, 5±4.1% of control snails (exposed to a GFP-specific siRNA oligo cocktail) shed cercaria, compared to 0% of the snails exposed to SmLeish knockdown parasites (Fig 5A). Statistically significant differences were observed between 5 and 7-weeks post challenge when 38±6%, 72.5±3% and 93±1.1% of control snails and 6.7±9.7%, 23±16.6% and 53.9±3.7% of SmLeish knockdown snails shed cercaria at 5, 6 and 7 weeks post challenge respectively. At 8 weeks post challenge and onwards the mean proportion of snails shedding cercaria in the SmLeish knockdown group caught up to the controls and both groups had similar percentages of snails shedding cercaria until 10-weeks post challenge; 94.9±3.2, 92±7.6 and 90±9 control snails shed cercaria and 75.3±11.5, 80.8±12.3 and 76.7±18.3 SmLeish knockdown snails shed cercaria (Fig 5A).

In two of the three SmLeish knockdown trials, the average number of cercaria that was shed by each shedding snail over a 24-hour time was also enumerated. Similar to the proportion of snails that shed cercaria, the number of cercaria shed was also impacted by knockdown of SmLeish. Control parasite exposure resulted in 14±5.7, 15.8±8.7, 27±10.7, 38.2± 15.4, 52.5±21.3, 70.3±28.3 and 76.9±29.5 cercaria released per shedding snail at 4, 5, 6, 7, 8, 9, and 10 weeks post challenge (Fig 5B). Whereas in snails exposed to SmLeish knockdown parasites 0, 9±0, 8.7±3.1, 12.8±7.9, 31.6±16.6, 46.7±28.3 and 65.6±28.6 cercaria were released per shedding snail at the same time points. Between 6 and 9 weeks post challenge, snails challenged by SmLeish knockdown parasites shed statistically fewer cercaria on average than the snails exposed to GFP knockdown *S*. *mansoni* (p<0.05) (Fig 5B).

### SmLeish KD *S*. *mansoni* are encapsulated *in vitro* by M-line *B*. *glabrata* haemocytes more quickly and frequently than GFP KD controls

The impact of SmLeish on *S*. *mansoni* infection kinetics may be mediated through an influence on miracidia penetration/establishment success and by delaying/preventing haemocyte encapsulation. Both visual and qPCR-based assessment of intramolluscan *S*. *mansoni* at day-2 post challenge suggest that fewer miracidia successfully penetrated and/or established within a M-line *B*. *glabrata* following knockdown of SmLeish (p<0.05) (S6 Fig). Visualization of fluorescently labelled *S*. *mansoni* miracidia/sporocysts within the snail head-foot of 30 M-line *B*. *glabrata* following exposure to 15 miracidia identified an average of 4.9±3.6 of SmLeish knockdown and 7.4±4.3 of GFP knockdown parasites (S6A Fig). Quantitative PCR analysis of a separate 30 M-line snails targeting the GAPDH gene of *S*. *mansoni* estimated that 5.3±0.8 and 6.9±0.7 successfully established in the GFP knockdown and SmLeish knockdown groups respectively (S6B Fig).

**Fig 6:**
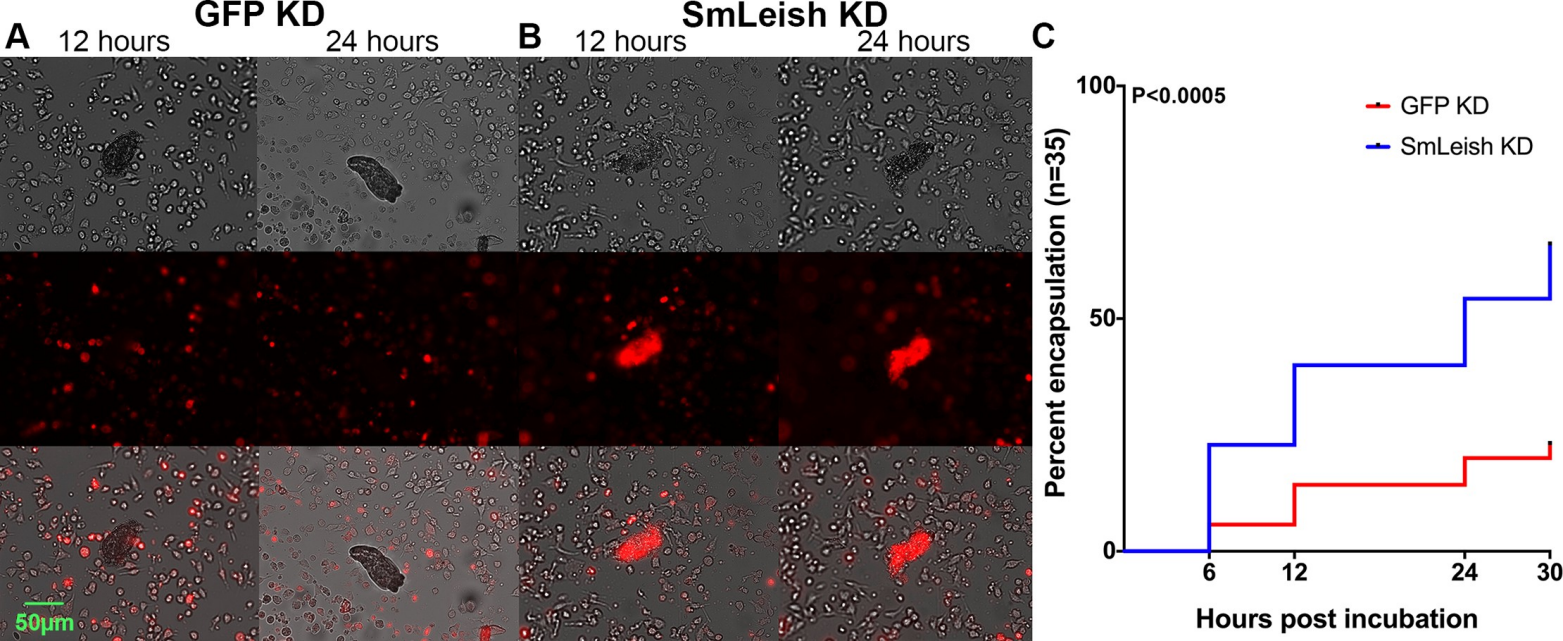
SmLeish impacts infection success by affecting the ability of haemocytes to encapsulate *S*. *mansoni* sporocysts. Encapsulation of *S*. *mansoni* sporocysts was visualized using fluorescently labelled M-line haemocytes and incubating them with GFP (A) or SmLeish (B) knockdown sporocysts that had been transformed *in vitro*. GFP knockdown control sporocysts in which SmLeish expression remains intact were infrequently encapsulated (A and C) compared to SmLeish-knockdown sporocysts, which were more frequently encapsulated by 24 hours post incubation (p<0.0005) (B and C). Representative images of an encapsulated and non-encapsulated sporocyst at 12 and 24 hours post incubation are shown (A and B).

The mechanism underpinning infection success in this case was hypothesized to be associated with the ability of haemocytes to be recruited and then ultimately encapsulate the sporocyst. Encapsulation kinetics were assessed *in vitro* using transformed *S*. *mansoni* sporocysts and isolated primary haemocytes from M-line *B*. *glabrata* labelled using a cell tracking fluorescent dye. Encapsulation of SmLeish-knockdown and control GFP-knockdown sporocysts was assessed at 6, 12, 24 and 30 hours post incubation by visualizing fluorescence around the sporocyst (Fig 6A and 6B). Out of the 35 sporocysts evaluated, 5.7%, 14.3%, 20% and 22.9% were encapsulated in the GPF knockdown control group compared to 22.9%, 40%, 54.3% and 65.7% encapsulated in the SmLeish knockdown group at 6, 12, 24 and 30 hours respectively (Fig 6C). The two infection curves were found to be significantly different using both a Log-rank (Mantel-Cox) test (p<0.0003), and a Gehan-Breslow-Wilcoxian test (p<0.0005). We ensured that we were observing differences in encapsulation rates rather than autofluorescence caused by dying parasites by examining miracidia and sporocysts unexposed to labeled haemocytes, as well as sporocysts exposed to unlabeled haemocytes, all of which failed to fluoresce (S7 Fig).

## Discussion

It is well documented that both host and parasite produce factors that can function as determinants of host-parasite compatibility. In the snail-digenean trematode model, numerous studies have demonstrated that molecules, present on the surface of the parasite or in their ES products, can influence or suppress various aspects of the snail immune response, ranging from pathogen recognition to effector responses [7, 32–35]. However, very little is known about the identity and mechanism of involvement of the specific parasite factors that underlie these effects. While molecules such as the *S*. *mansoni* polymorphic mucins provide insight into how the parasite might evade snail immune recognition and response [36], almost nothing is known about parasite factors that dampen the snail immune response to facilitate infection establishment. In an effort to identify specific factors underpinning these effects, we have functionally characterized an *S*. *mansoni* metalloprotease that shares amino acid and predicted structural similarities to leishmanolysin. Functional assessment of this MMP, SmLeish, suggests that it is an important parasite-produced suppressor of the snail cellular immune response, impacting haemocyte migration and ultimately influencing parasite encapsulation and *S*. *mansoni* infection kinetics.

Recombinant SmLeish clearly exhibited dose-dependent MMP activity. The enzymatic activity demonstrated by the non-trypsinized recombinant suggests rSmLeish was produced in a form that was partially active despite a lack of proteolytic cleavage by any host or parasite factor. Treatment of rSmLeish with mammalian trypsin did, however, result in higher levels of MMP activity, suggesting that rSmLeish may have been activated due to non-specific removal of its N-terminal region. This finding was expected given that many metalloproteases require cleavage of their N-terminal ends in order to become functional [21]. This does not necessarily lead us to believe that trypsin is the most biologically relevant activating enzyme for SmLeish, but rather that trypsin is one of many possible enzymes that can generate a more active form of SmLeish. Even MMP-8, our positive control for this experiment, can be activated by an array of different enzymes [37–39]. When treated with the human MMP inhibitor Ilomastat, both trypsinized and non-trypsinized forms of rSmLeish had reduced MMP enzymatic activity. Inhibition of rSmLeish was more evident when a different MMP inhibitor, 1,10-phenanthroline, was applied. This inhibitor has been shown to inhibit the function of leishmanolysin [40], and perhaps more relevantly, has also been shown to negatively affect the viability, motor activity and fecundity of adult *S*. *mansoni in vitro* [41], as well as inhibit *S*. *mansoni* leucine aminopeptidase activity [42]. These data support the hypothesis that SmLeish is functioning as a MMP, thereby providing a foundation for understanding the phenotypic changes regarding parasite encapsulation observed in both this work and previous studies [7,8].

A role for SmLeish in host immune evasion during initial establishment within the snail is additionally supported by both the increase in transcript levels and protein expression observed throughout infection. The relative increase of SmLeish transcripts peaking at 12 hours post infection, coupled with the appearance of the ~48kDa SmLeish form in infected snail haemolymph at the same time confirms the presence of SmLeish during initial establishment and infection stages. This quick upregulation, as well as the observation that SmLeish is also present in the snail plasma early in the infection suggests a role in evading haemocyte encapsulation, which typically occurs within the first 48 hours post infection [43]. Additionally, since SmLeish-like genes are present in non-parasitic flatworms that would not encounter a host immune response, it is also likely that the observed upregulation of SmLeish is also linked to the large amount of tissue remodeling occurring during the transition from miracidium to sporocyst [44]. This is consistent with the idea that parasitism is an evolved trait amongst flatworms [45] and suggests that SmLeish may have functions outside of those described here in relation to facilitating infection.

Of interest was the observation that a relative increase in transcript levels was not restricted to the early stages of infection, but was also evident at 35 days post infection, which correlates with cercarial shedding under our exposure conditions [46, 47], and in isolated cercariae. Coupled with the observation that leishmanolysin-like proteins have been previously identified in schistosome cercarial secretions, this supports the hypothesis that SmLeish may also function in immunosuppressive activities during the initial stages of tissue penetration and schistosomula migration in the human hosts [12, 48, 49]. To add further to this, Verjovski-Almeida et al. (2003) demonstrate that the transcript for SmLeish is expressed by adult *S*. *mansoni*, suggesting a possible role in facilitating survival within the human host as well [50].

We observed a ~130kDa form of SmLeish at 1-day post infection, association of this protein with *S*. *mansoni* sporocysts was confirmed via immunofluorescent detection. Additionally, a soluble ~48kDa variant which is expected to be the result of cleavage, was located in snail haemolymph. To determine whether this protein was the result of alternative splicing, three RT-qPCR assays designed to span the SmLeish transcript were performed and returned statistically similar results. Thus, it is unlikely that alternative splicing is responsible for the appearance of the 48kDa protein as one of these assays would have been expected to coincide with the appearance of the ~48kDa band. Both observations agree with the existing leishmania literature suggesting leishmanolysin exists in a full-length form, as well as a cleaved form secreted into the surrounding environment. The origins of this soluble form of SmLeish remain unknown, but research into leishmanolysin suggests it may result from both direct secretion and cleavage of the membrane bound pro-leishmanolysin [18, 51]. Our MMP activity assay results suggest that both forms of SmLeish are functionally active, although the full-length recombinant we generated lacked part of the N-terminal end of the protein, thereby disallowing us to conclusively state that the membrane bound form of SmLeish demonstrates MMP activity. Indeed, the majority of MMPs, including leishmanolysin, require proteolytic cleavage of their N-terminal end to become active [18, 21]. Trypsin treatment of rSmLeish did produce two smaller proteins that tandem MS analysis mapped to the C-terminal end of the full-length SmLeish protein. This suggests that the higher MMP activity and slightly more substantial decrease in M-line haemocyte motility displayed by the trypsin treated rSmLeish compared to the non-trypsinized rSmLeish may be due to the presence of the smaller (soluble) SmLeish.

The haemocyte migration assay allowed us to draw two important conclusions regarding haemocyte motility and the function of SmLeish. The first is that SmLeish negatively impacts the ability of haemocytes to migrate, with this effect proving increasingly potent with M-line haemocytes relative to those from BS-90 snails. The second being that BS-90 haemocytes are attracted to sporocyst ES products, while M-line haemocytes are not. The observation that treatment of BS-90 haemocytes with rSmLeish does not result in the same chemoattractant response as treatment with sporocyst ES products suggests that SmLeish is not the chemo attractive component that BS-90 haemocytes respond to. This observation is confirmed by the fact that ES products from SmLeish knockdown *S*. *mansoni* are still attractive to BS-90 haemocytes. Seeing as treatment with ES products lacking SmLeish (via immunoprecipitation, 1,10-phenanthroline pretreatment or knockdown of SmLeish) resulted in a return of M-line haemocyte mobility to baseline levels, we predict that SmLeish is playing a significant role by dampening haemocyte recruitment to the sporocyst within the snail host during *S*. *mansoni* infection.

It is not yet possible to make any conclusions regarding the mechanism of haemocyte migration inhibition. Cell movement towards a target is dependent upon sensing chemical gradients, adhesion to a substrate, and remodeling of the cytoskeleton, rendering these pathways possible targets. Targeting of SmLeish to these substrates is supported by the knowledge of leishmanolysin targeting myristoylated alanine-rich C kinase substrate-related proteins in humans, which are involved in cell signaling [52], and the fact that many MMPs target proteins comprising important parts of the cytoskeleton, such as collagen [26, 53]. Another potential target, due to the ability of leishmanolysin to cleave fibrinogen [21], would be the fibrinogen domain of the *B*. *glabrata* Fibrinogen-Related Proteins (FREPs), which are a unique group of molecules that play a role in parasite recognition and opsonization of sporocyst tegument-associated carbohydrates [54–56]. Additionally, the observation that leishmanolysin assists in the avoidance of complement mediated lysis of Leishmania through the cleavage of C3b [22] suggests that the snail thioester-containing protein may also be one of the targets of SmLeish. What we can conclude is that the negative impacts of SmLeish on M-line haemocyte migration are likely due to the MMP enzymatic activity (supported by the fact that rSmLeish is inhibited by 1,10-phenanthroline), and that the negative effect of ES products is likely because of SmLeish being present (supported by the ES+1,10-phenanthroline and the fact that SmLeish knockdown abrogates the effect and can be rescued by addition of rSmLeish).

Regardless of what the target of SmLeish may be, questions remain as to why this MMP fails to impede the mobility of BS-90 haemocytes, while reducing the mobility of M-line haemocytes. It is possible that allelic differences in the SmLeish target between these strains result in an inability of SmLeish to effectively hydrolyze the target in susceptible snails but not in resistant ones. Alternatively, such allelic differences may not be present in potential SmLeish targets, but rather in snail-associated pattern recognition receptors responsible for the targeting of and subsequent neutralization of SmLeish.

The utilization of siRNA mediated knock-down of SmLeish proved successful in reducing levels of the protein during infection. While snails infected with GFP KD miracidia still demonstrated a rise in SmLeish transcription levels peaking at four days post infection, those infected with SmLeish KD miracidia featured no such increase, confirming specific targeting of the SmLeish mRNA. While this does not control for the possibility of off-target impacts of the administration of the siRNA oligos, the inclusion of the GFP-specific siRNA control accounts for the possibility that any off-target effects might impact the outcome of the encapsulation or chemokinesis bioassays [57]. Protein expression was also affected by the siRNA treatment, with the soluble ~48 kDa variant becoming undetectable in whole snail lysates, while the ~130 kDa variant exhibited a decrease in expression at 4 days post infection. The complete absence of the soluble ~48 kDa variant is possibly the result of a reduction in the amount of the full length ~130 kDa protein, resulting in less activating cleavage events.

GFP KD sporocysts were able to avoid encapsulation by M-line snail haemocytes more effectively than SmLeish KD sporocysts. Although both treatments resulted in sporocysts being encapsulated as early as 6 hours post infection, the percentage of encapsulated sporocysts was consistently higher in the SmLeish KD group until the experiment was terminated. After 30 hours, 65.7% of SmLeish KD and only 22.9% of GFP KD sporocysts had been encapsulated. These results are also consistent with the work of Joshi et al. (2002), which demonstrate that targeted gene deletion of Gp63 in *Leishmania major* results in a delay in lesion formation in its mouse host, a process reversed by the introduction of a functional copy of Gp63 [58]. Although our model system does not possess the advantage of full deletion of SmLeish, the increased frequency with which our SmLeish KD sporocysts are encapsulated by the snail immune response, and the abrogation of the negative migration effect of SmLeish in knockdown ES products draws parallels to *L*. *major* Gp63 gene deletion mutants, which were more effectively eliminated by the mouse immune response. This is suggestive that in both parasites, leishmanolysin functions as a key component during the interactions between the parasite and the host immune response and appears to function in favour of the parasite [58].

That SmLeish plays role in facilitating *S*. *mansoni* establishment within the snail host was further supported by the changes in the time it took for infections to reach patency, and the lowered cercaria output when SmLeish KD miracidia were used to challenge snails. Both phenotypic changes were likely caused by a reduction in the number of parasites that could successfully evade the haemocyte-mediated immune response, seeing as lower numbers of parasites per infection were confirmed using both visual inspection and qPCR. The lack of a complete abrogation of patent infections suggests that although SmLeish plays a significant role in determining infection outcome, *S*. *mansoni* likely utilizes other means by which it dampens the ability of the snail immune response to recognize and eliminate the invading larval parasites. Alternatively, the presence of the pro-protein that remained associated with the SmLeish KD sporocysts may have retained enough function to prevent encapsulation in some cases.

Parasites are master immune modulators that utilize multiple methods of ensuring their survival inside of their hosts. *S*. *mansoni* proves an excellent example of this, as different life stages employ different immunomodulatory mechanisms which allow it to infect, survive, and reproduce in two vastly different hosts [59–61]. Our work successfully demonstrates that SmLeish functions as an MMP and supports a role in facilitating sporocyst survival within the snail by impeding the encapsulation response, resulting in increased infection success, kinetics and output. Future work should strive to discover the specific targets of SmLeish on *B*. *glabrata* haemocytes, while also seeking to discover the method by which it is activated during the intramolluscan stages of *S*. *mansoni* infection. Identifying the specific active site in SmLeish would allow for the synthesis of inactive mutants that could concretely demonstrate that the immunomodulatory roles of this MMP are due to enzymatic activity. The functional characterization of SmLeish described here provides an insightful and necessary step in understanding the schistosome/snail relationship, which serves to both deepen our knowledge of the intricacies of invertebrate immunology and will ultimately allow us to examine possible methods of reducing the worldwide prevalence and impact of schistosomiasis.

## Materials and methods

### Ethics statement

All animal work observed ethical requirements and was approved by the Canadian Council of Animal Care (AUP00000057).

### Snails and parasites

Two strains of *B*. *glabrata* snails were used in this study. The BS-90 strain is resistant to PR-1 strain *S*. *mansoni* infection [62, 63], while the M-line strain is susceptible [64]. Snails were maintained in aerated artificial spring water at 23–25°C, 12-hour day/night cycle and fed red-leaf lettuce as needed. All snail exposures were performed with the PR-1 strain of *S*. *mansoni* [64], which was obtained from infected Swiss-Webster mice provided by the NIH/NIAID Schistosomiasis Resource Center at the Biomedical Research Institute [65].

### Identification and *in silico* analysis of SmLeish

Transcripts and proteins resembling leishmanolysin of *Leishmania major* [18, 66] have been identified in numerous screens of schistosomes at varying life history stages including during the human infection [9–12, 48, 67] and snail [13–15] hosts. Improvement of the *S*. *mansoni* genome [68], identified two complete leishmanolysin-like transcripts, one of which (XP_018651919), possessed two predicted M8 protease domains (http://pfam.xfam.org). This transcript was used for all *in silico* analyses and served as a basis for all functional assessments.

The predicted amino acid sequence for SmLeish (XP_018651919.1) was assessed using the program Phobius [69] to determine that SmLeish is likely a non-cytoplasmic protein with a weak signal peptide (residues 1–20) with a cut site between positions 20 and 21 as assessed using SignalP 4.1 [70].

### Sporocyst transformations and isolation of *S*. *mansoni* excretory/secretory products

Mice infected with *S*. *mansoni* were euthanized 7 to 8 weeks post exposure and their liver extracted and homogenized using an Omni Mixer Homogenizer model N^0^17105 for 60 seconds. The homogenized product was added to a 2L flask filled with artificial spring water. The flask was covered in aluminum foil except the top 5 cm of the flask. Light was shone at the top uncovered part of the flask to encourage the migration of newly hatched miracidia to the top of the flask thus facilitating their subsequent isolation.

After 24 hours culture in Chernin’s Balanced Salt Solution (CBSS) [71] containing glucose and trehalose (1g/L each), penicillin (100 U/mL) and streptomycin (100 μg/mL), most miracidia transformed to primary sporocysts. Parasite culture supernatants containing ES products [13] were collected and sterilized with a 0.2 μm syringe filter, concentrated and stored at −80°C. Sporocysts were collected in two separate conditions: first from regular *S*. *mansoni* to obtain ES products, and second from SmLeish knockdown *S*. *mansoni* miracidia which were later used to evaluate the success of the knockdown. Excretory/secretory products were collected in the same way as described above for SmLeish knockdown and GFP knockdown *S*. *mansoni*. In these cases, knockdown was performed as described below during sporocyst transformation, ES products were collected following knockdown in fresh medium.

Primary sporocysts from SmLeish knockdown miracidia were kept for 5 days in conditioned complete *B*. *glabrata* embryonic (Bge) cell medium prepared from culture supernatants of 4-day maintained Bge cells as previously described [72, 73]. Sporocysts were collected every day until day 5 post challenge. RNA was purified and underwent reverse transcription immediately after collection of the sporocysts so that later analysis of SmLeish transcription could be undertaken to confirm knockdown efficiency.

### Production of recombinant SmLeish

Recombinant SmLeish was generated by using the Gateway cloning system according to the manufacturer’s instructions (Life Technologies). The coding region was amplified with Phusion high-fidelity DNA polymerase from a targeted DNA template in a pUC57 plasmid (synthesized by GenScript) and cloned into the pENTR/D-TOPO vector. Plasmid DNA from this entry clone was isolated and cloned into the pET-DEST42 Gateway vector (Life Technologies) in a Clonase recombination reaction. This DNA was then transformed into BL21-AI One Shot Chemically Competent *E*. *coli* (Life Technologies).

*E*. *coli* stably expressing SmLeish were then selected and grown up at 37°C in 100μg/mL ampicillin LB medium. Optimal expression of protein after the incorporation of L-arabinose and IPTG was then quantified by SDS-PAGE and Western blot using antibodies against the 6xHis tag and V5 epitope (Life Technologies) on the recombinant protein.

Prior to purification of the recombinant protein, *E*. *coli* were concentrated by centrifugation at 10 000 rpm for 20 minutes at 4°C. The final weight of the bacteria pellet was measured and then the lysing reagent B-PER (Thermo Fisher Scientific) was added at a concentration of 4mL/g of bacteria in combination with phenylmethylsufonyl fluoride (PMSF, final concentration of 1mM), mixed gently and left to incubate at room temperature for 15 minutes. After the incubation, the samples were centrifuged at 10 000 rpm for 10 minutes and the supernatant kept for the purification steps. Before application to the 6xHIS column for purification, the supernatant was diluted to a total protein concentration of 100μg/mL in binding buffer (GE Healthcare). Fast protein liquid chromatography (FPLC) (AKTA Pure–GE Healthcare) was used to purify rSmLeish protein using 1mL nickel-agarose columns that bind to the 6xHis region of the recombinant protein (GE Healthcare). Prior to quantification via BCA protein quantification assay (Thermo Fisher Scientific), the purified rSmLeish was dialyzed using a Slide-A-Lyzer Dialysis Cassette kit (Thermo Fisher Scientific) as per the manufacturer’s instructions (S3 Fig).

### Generation of an anti-SmLeish polyclonal antibody

Rabbit anti-SmLeish polyclonal antibodies were generated using engineered peptides with the sequence EEDGTPRTPRDPQT (GenScript) predicted to have a high level of antigenicity based OptimumAntigen design tool (GenScript). Serum IgG was purified using a Protein A/G column (GE Healthcare) by FPLC. Further purification was undertaken by then running the purified IgG through an immunoaffinity column bound with the specific peptides to which the polyclonal antibody was designed.

The specificity of the polyclonal antibody was tested during a dot blot test against its cognate peptide antigen and was also tested against recombinant SmLeish as well as *S*. *mansoni* ES products (S3 Fig and Fig 4 respectively). The antibody was also used to measure the approximate concentrations of SmLeish in *S*. *mansoni* ES products and 2-day post challenge M-line *B*. *glabrata* (n = 5).

### Estimation of SmLeish in *S*. *mansoni* ES products and snail plasma

ES products and M-line cell-free plasma was collected as described above and plasma was diluted 1/10 in 0.2M NaHCO_3_ (pH 9.4) prior to addition of 100μL into a 96-well polystyrene ELISA plate. Plates were incubated at 4°C for 24 hours and then washed 3x 5 minutes with wash buffer (25mM Tris, 0.15M NaCl, 0.05% Tween 20, pH 7.2). Plates were then blocked with 2% (w/v) bovine serum albumin in wash buffer for 3 hours at room temperature. Following blocking, the buffer was replaced with 100μL of blocking buffer containing anti-SmLeish antibody (1:500) and incubated at room temperature overnight. Plates were then washed 3x 5 minutes with wash buffer and then incubated with blocking buffer containing a biotinylated anti-rabbit secondary antibody (1:250). Plates were then covered with tin foil and incubated at room temperature for 1 hour, washed 6x 5 minutes, and then incubated in the substrate solution containing streptavidin conjugated to DyLight 649 (Thermo Scientific). The reaction proceeded for 15 minutes and was then read using a 96-well plate reader (Molecular Devices). All plasma samples were compared to plasma samples collected from non-challenged M-line *B*. *glabrata* (n = 3) and a standard curve generated using a serial dilution series of rSmLeish ranging from 0.0625μg/mL to 2μg/mL. Control snail plasma yielded a slight background signal that translated into an estimated 541±159pg/mL SmLeish. The average estimated SmLeish in the plasma of the five *S*. *mansoni-*challenged *B*. *glabrata* was 27,778±22,342pg/mL. This group displayed a vast range of values with the highest being from a snail in which the estimated SmLeish was 68,804pg/mL. SmLeish was estimated to be present at 106,829±4944pg/mL in *S*. *mansoni* ES products. For reference, the 0.25μg/mL rSmLeish yielded estimated SmLeish concentrations of 230,275±5223pg/mL (S8 Fig). Thus, the lowest rSmLeish concentration used in our experiments reflects about 2.5x more than is found in the ES products.

### Immunofluorescent detection of SmLeish during intramolluscan *S*. *mansoni* development

*S*. *mansoni*-infected *B*. *glabrata* were frozen in Tissue-Tek OCT (VWR) and cryosectioned in 7μm sections. Sections were transferred to poly-L-lysine microscope slides (Abcam). Slides were then washed twice in TBS and 0.025% Tween-20 solution and blocked using 10% FBS with 1% BSA in TBS for two hours at room temperature. The primary antibody (anti SmLeish or Keyhole limpet hemocyanin (KLH)) diluted in TBS with 1% BSA at a concentration of 1:250 was added to the slides and incubated overnight at 4°C in a humidified chamber. Slides were then rinsed twice for five minutes in TBS and 0.025% Tween-20 with gentle agitation. The secondary fluorophore-conjugated antibody (Alexa Fluor 488) diluted in TBS with 1% BSA following the manufacturer’s recommendations was added to the slides and incubated at room temperature for one hour. In the dark, slides were rinsed three times in TBS for five minutes each and one drop of DAPI mounting medium (VWR) was added to the specimen. After five minutes, a coverslip was placed over the mounted tissue. Slides were imaged using an Axio imager A2 microscope (Zeiss), and analyzed using Zen 2011 software (Zeiss) and Photoshop CS5 (Adobe Systems Inc., USA).

### Evaluation of SmLeish metalloprotease activity

Functional characterization of rSmLeish was performed using the Sensolyte Generic MMP Colorimetric Assay (Anaspec). Following manufacturer’s directions, rSmLeish was incubated with the provided chromogenic substrate that is cleaved by MMPs. The sulfhydryl group reaction with Ellman’s reagent yields 2-nitro-5-thiobenzoic acid (TNB) as the final product, which is detected using a microplate reader at 412 nm. Human MMP-8 obtained from Anaspec was used as a positive control. Three initial concentrations (0.25 μg/mL 0.5 μg/mL and 1μg/mL) of the enzymes were used. Human MMP-8 and rSmLeish were both exposed to 10 μg/mL trypsin for one hour at 37°C as an activation process as per the MMP-8 assay instructions that was stopped using a trypsin inhibitor (Anaspec). Recombinant SmLeish was also exposed to 5 μg/mL of trypsin for 0, 6 and 12 hours to determine whether longer exposure influenced trypsin activation or rSmLeish cleavage. The inhibition of the human MMP-8 and rSmLeish activity was assessed by adding the MMP inhibitor Ilomastat (MMP8 and rSmLeish) or 10nM 1,10-phenanthroline (rSmLeish) to the initial enzyme before adding the substrate. These reactions were performed in 96-well plates, and the OD_412_ values were obtained using a SpectroMax M2 fluorescent plate reader. These values were compared to a standard curve generated using a reference provided by Anaspec. Significant differences between treatments were assessed by one-way analysis of variance (ANOVA) with Tukey’s post-hoc test.

### Quantifying SmLeish transcript and protein abundance during the intramolluscan development of *S*. *mansoni*

#### Transcript abundance

Using TRIzol Reagent with the PureLink RNA Mini Kit (Life Technologies) and following the instructions of the manufacturer, RNA from each snail was isolated and then stored at -80°C. Complementary DNA (cDNA) was generated using this total RNA with the Quanta Biosciences qScript cDNA SuperMix Kit according to the manufacturer’s instructions.

Quantitative RT-PCR primers and probe for SmLeish were designed using the qPCR assay design tools from Integrated DNA Technologies (IDT) (Table 1). Three unique qPCR assays for SmLeish were designed and utilized to assess whether alternative splicing was taking place and to ensure that assessment of transcript abundance was yielding consistent results across the entire transcript. The SmLeish qPCR reaction consisted of 10μL of TaqMan mix (Thermo Fisher Scientific), 0.4μL of the forward and reverse primers, 0.2μL of the probe, 4μL of nuclease free water, and 5μL of SmLeish template. The qRT-PCR reactions were run in triplicates in 96-well qRT-PCR plates for 40 cycles on an ABI 7500 Fast Real Time PCR machine (Thermo Fisher Scientific) using the following thermocycling conditions: initial hold at 95°C for 10 minutes, followed by 40 cycles of 95°C for 15 seconds and 60°C for 1 minute, with data collection every cycle. Specificity for the qRT-PCR amplicons was confirmed by continuous melt curve analysis. Significant differences between treatments were assessed by one-way analysis of variance (ANOVA) with Tukey’s post-hoc test.

To confirm that challenged snails used in the SmLeish transcript expression analysis were infected with *S*. *mansoni*, *S*. *mansoni-*specific GAPDH was assessed using an established assay and protocol [47, 74]. This assay was also used to estimate the number of *S*. *mansoni* miracidia (n = 30 SmLeish-KD and 30 GFP-KD) that had successfully penetrated *B*. *glabrata* snails by comparing Ct values to a standard generated using known numbers of *S*. *mansoni in vitro-*transformed sporocysts (S4 Fig).

#### Protein assessment

Recombinant SmLeish or *S*. *mansoni* ES products were separated by SDS-PAGE under reducing condition using 12% polyacrylamide gels, which was then transferred to a 0.45μm nitrocellulose membrane (BioRad). Following blocking in 5% milk in TBS-T buffer (1% v/v tween-20), the membrane was incubated overnight in primary antibody (1:5000) in blocking buffer (5% milk in TBS-T). Membranes were washed 3x in TBS-T, prior to a 1-hour incubation in secondary antibody (1:5000 in TBS-T). Finally, 3 washes in TSB-T and then 3 additional washes in TBS were undertaken prior to development. To develop the membrane, an ECL Western blotting detection kit (GE Healthcare) following the manufacturer’s descriptions was used and an ImageQuant LAS400 detection instrument was used to visualize following the manufacturer recommendations. For both *B*. *glabrata* whole snail lysate and plasma, ~30μg of total protein was loaded into each well of the SDS-PAGE. Because no appropriate loading control exists for snail plasma, total plasma amount loaded normalizes the Western blot. For total snail lysates, ß-actin was used as a loading control.

### Assessing the impact of rSmLeish on haemocyte movement

The impact of rSmLeish and *S*. *mansoni* ES products on *B*. *glabrata* haemocyte migration was assessed using a chemokinesis assay that has been previously published [32]. This approach does not directly measure chemotaxis towards a target gradient, but instead measures the impact of a molecule of interest on cell migration behaviour compared to controls. This assay was chosen because we believed SmLeish to be inhibitory to chemokinesis but did not have any available *S*. *mansoni*-specific targets known to be chemo attractive to *B*. *glabrata* haemocytes. Also, because the specific target of SmLeish activity is not known, we were unsure of how it would affect chemotaxis induced by a known factor.

The assay was designed to test the impact of both rSmLeish and *S*. *mansoni* ES products on haemocyte migration of both M-line and BS-90 strains of *B*. *glabrata*. BS-90 snails are resistant to *S*. *mansoni* infection, and thus served as a control for the infection phenotype and the hypothesized role of SmLeish in facilitating infection establishment in M-line snails. In all tests, haemolymph was isolated from an individual snail, centrifuged at 500 X g for 10 minutes, and the haemocyte pellet was isolated by aspirating off the cell-free plasma. The haemocytes were resuspended in 200μL of 1x PBS (2.58 mM NaH_2_PO_4_, 7.68 Na_2_HPO_4_, 150 mM NaCl, pH7.4) and then divided into two equal volumes. Each subset of haemocytes was counted using a haemocytometer (10μL) to ensure equal cell concentrations in each subset. This was done in replicates of five snails per treatment, per strain.

Haemocyte chemokinesis was measured using a custom-built chemotaxis chamber. Haemocytes were always placed into the upper chamber of the apparatus, which is separated from the lower chamber by a 5μM pore-containing membrane. All analyses consisted of staining the membrane with hematoxylin and eosin (H and E) and then counting the number of haemocytes that migrate to the underside of the membrane. In each case, the experimental test was compared to a snail-matched control which consisted of the second haemocyte subset of each snail isolation. Controls were exposed to CBSS in both the upper and lower chambers of the apparatus and thus represented the baseline haemocyte migration (chemokinesis) for each snail.

The experimental groups used to test SmLeish included: 0.25μg/mL rSmLeish (which was demonstrated to have enzymatic activity as a MMP and represents approximately 2.5x more SmLeish than is found in our ES products) and 0.5μg/mL *S*. *mansoni* ES products. 1μM fMLP was used as a positive control. Recombinant SmLeish, *S*. *mansoni* ES, rSmLeish and ES products with SmLeish immunoprecipitated prior to treatment, rSmLeish incubated with 10μg/mL trypsin for 1 hour prior to treatment, rSmLeish incubated with 10nM 1,10-phenanthroline prior to treatment, fMLP contrasted to ES products or rSmLeish, SmLeish and GFP knockdown ES products and SmLeish knockdown ES products rescued with 0.25μg/mL rSmLeish were assessed for their impact on haemocyte migration in three ways: first by placing the agent in the bottom well to assess attractiveness to haemocytes, second by placing the agent in the top well with the haemocytes to assess inhibition of chemokinesis, and finally, the agent was placed in both wells, which is a traditional control for chemotaxis to assess baseline chemokinesis. Each experimental set-up was replicated five times with independent snails supplying haemocytes for each experiment. The primary experimental groups were all run with each possible combination of upper, lower and both chambers including the treatment. In the case of any treatment that includes trypsin, the treatment was inactivated using Trypsin Neutralizing Solution (ATCC) prior to incubation with haemocytes.

### Knockdown of SmLeish using siRNA

*In vitro* transformed *S*. *mansoni* sporocysts were submerged in a 6nM cocktail of 27-nucleotide siRNA oligonucleotides (Integrated DNA Technologies) designed to specifically target 4 different regions of the SmLeish transcript. The oligonucleotide sequences were confirmed to be unique to SmLeish by comparison to the *S*. *mansoni* genome. As has been previously reported [75, 76], we found that soaking the sporocysts in a cocktail of siRNA oligos resulted in knockdown of SmLeish, this was made more consistent by using the Xfect transfection reagent (Clone Tech).

Control sporocysts received siRNA oligo targeting green fluorescent protein (GFP) (Table 2). Confirmation of SmLeish knockdown was accomplished using the pre-existing qRT-PCR assay described above. RNA was isolated from *S*. *mansoni* sporocysts at 1, 2, 3, 4, and 5-days post exposure to either the SmLeish or GFP-specific siRNA oligos for 24 hours. Transcription of SmLeish *in vitro* during this period was assessed and compared to sporocysts exposed to GFP-specific siRNA. In each case, 10 sporocysts were used to generate pooled total RNA from which cDNA for qRT-PCR was synthesized as described above (S5 Fig).

**Table 2:** siRNA oligonucleotide sequences.

Target	Oligonucleotide sequence
siRNA-SmLeish-1	5ʹ- GAAGCUUUCAGAUAUAUGCGAGACCAG -3ʹ
siRNA-SmLeish-2	5ʹ- CGAACUGUAGAUGCUUUUGUGCUUAUA -3ʹ
siRNA-SmLeish-3	5ʹ- UUACCUACCGCCUUCCGAAUUCGCCAG -3ʹ
siRNA-SmLeish-4	5ʹ- UCGAAUCUUGUUAAACUCUACCUUCUA -3ʹ
siRNA-GFP-1	5ʹ-CCAUCAUCUUUGAAGAAGGAACAAUCUUCUUCAAAG-3ʹ
siRNA-GFP-2	5ʹ-AGGUAAUAAUACAGGACCCGGUGAUGGUCCUGUAUU-3ʹ
siRNA-GFP-3	5ʹ-AUGUUGUUACUAAUGUAGCCUUGACCUACAUUAGUA-3ʹ

Fifty M-line *B*. *glabrata* were challenged with *S*. *mansoni* miracidia exposed to either SmLeish or GFP-specific siRNA oligos 24 hours prior. Snails were exposed for 24 hours before being placed into tanks containing artificial spring water (25 snails per tank) and were fed biweekly a diet of red leaf lettuce. Beginning at 4-weeks post challenge and continuing subsequently every week, snails were placed in 24-well plates individually and incubated for 24 hours to assess cercaria shedding. Snail mortality was also noted. The experiment culminated at week 10 post challenge. Data was assessed as a percentage of snails shedding cercaria. Statistically significant differences in the proportion of snails shedding cercariae and the number of cercariae shed was determined using a z-test with a significance threshold of p<0.05.

### Quantifying the impact of SmLeish knockdown on sporocyst encapsulation

Quantifying *S*. *mansoni* sporocyst encapsulation by *B*. *glabrata* haemocytes was accomplished by fluorescently labeling primary haemocytes and incubating them for up to 30 hours with transformed sporocysts *in vitro*. Sporocyst transformation was performed as has been previously described [13], and primary haemocytes were isolated using the head-foot retraction method [46]. Prior to sporocyst transformation, the miracidia were exposed to siRNA targeting either SmLeish or GFP as described above. Haemocytes from five snails were pooled and counted using a haemocytometer. Prior to incubation with sporocysts, haemocytes were labeled using CellTracker Red CMTPX (Thermo Fisher Scientific) following the manufacturer’s instructions. Following incubation, haemocytes were washed using CBSS 5x for 10 minutes each, and then resuspended in CBSS at a final concentration of 100 cells/μL. Ten microliters of the pooled haemocytes were incubated with five transformed sporocysts (either SmLeish or GFP knockdown) in a depression well microscope slide. Every hour starting from the moment haemocytes and sporocysts were co-incubated, images of each sporocyst were captured using bright field and fluorescent microscopy. In total, 35 individual sporocysts in each group were tracked and imaged at each time point. Detection of the fluorescently labeled haemocytes allowed for visualization of the encapsulation response and later quantification of the number of sporocysts encapsulated at four selected time points, 6, 12, 24 and 30 hours post incubation. The resulting encapsulation time course was visualized by treating the data as an infection study and the two encapsulation curves were analyzed using Graphpad Prism version 7a for Mac (GraphPad Software) using both a Log-rank (Mantel-Cox) test, and a Gehan-Breslow-Wilcoxian test.

## Supporting information

S1 Fig SmLeish transcript abundance measured using three amplified regions. RT-qPCR results do not vary in a statistically significant manner at any time point among three separate sets of primers and probes used to assess the transcript abundance of SmLeish. The primer/probe combination in panel A was used for further analysis such as knock down efficiency, while the primer/probe combinations in panel B and C serve to rule out alternative splicing.
(TIF)

S2 Fig SmLeish possesses hallmark MMP domains. A) SmLeish features a predicted Leishmanolysin-like Peptidase/Metalloprotease (zincins) catalytic domain, which is in turn composed of two predicted Peptidase M8 domains. Within each of these domains resides the canonical HEXXH motif (blue) found in all zinc metalloproteases, which are components of the HEXXHXXGXXS motifs (green) seen across Metzincin Metalloproteases. B) The amino acid sequence of SmLeish highlights the presence of two Peptidase M8 domains and their respective active site motifs. The peptide sequence against which the anti-SmLeish antibody was derived is indicated by the black band.
(TIF)

S3 Fig Recombinant SmLeish is cleaved by trypsin. A) SDS-PAGE of the purified rSmLeish demonstrating a pure sample. B) Recombinant SmLeish cleaved using 5ug/ml trypsin for 0, 6, and 12 hours demonstrate the occurrence of a ~48kDa cleavage product, which increases in abundance following longer incubation times. C) Western blot of purified rSmLeish (rSmL) and rSmLeish following a one-hour incubation with 1, 5 and 10μg/mL trypsin at 37C. Cleavage products were detected at ~50kDa and ~35 kDa, as well as at ~25 and 20kDa. MS/MS analysis of the 50 and 35kDa cleavage products indicates that they possess peptide fragments of SmLeish (SmLeish peptide containing products indicated by arrows).
(TIF)

S4 Fig Controls for haemocyte migration. Additional dual well and trypsin/1,10-phenanthroline treatment controls for the haemocyte migration assay. Additionally, immunoprecipitation (IP) of SmLeish from ES products or out of the rSmL treatments provides further evidence that SmLeish is negatively impacting M-line haemocyte motility across the 5μm pore membrane. * indicate significant difference between indicated treatment and the non-SmLeish immunoprecipitated treatment shown in Fig 4A.
(TIFF)

S5 Fig siRNA-mediated knockdown of SmLeish. Assessment of *in vitro* transformed *S*. *mansoni* sporocyst expression of SmLeish following SmLeish knockdown (red bars) was compared to GFP knockdown controls (blue bars). Knockdown was significant at the transcriptional level as early as 2 days post incubation and prevented increases in transcript abundance that are traditionally observed for SmLeish by 3 days post transformation (A). Western blot analysis confirmed protein-level knockdown occurs as well, but is less evident for the larger SmLeish protein, while the ~48kDa soluble form of SmLeish is not detectable following knockdown (B).
(TIF)

S6 Fig Infection success of SmLeish knockdown *S. mansoni* is reduced compared to knockdown controls. Labeling of the *S*. *mansoni* miracidia using a membrane fluorescent dye allowed us to visualize the larval parasites within the head-foot of challenged M-line *B*. *glabrata*. This approach enabled us to count the number of sporocysts within the head-foot at 4 days post challenge using 15 labelled miracidia. A) Fewer SmLeish knockdown parasites were observed via visual examination within the head-foot on average compared to GFP knockdown controls. B) qPCR results also suggested a decrease in the among of SmLeish knockdown parasites present within the snail when compared to GFP knockdown controls.
(TIF)

S7 Fig Haemocyte encapsulation and fluorescence controls. *S*. *mansoni* larvae fail to autofluorescence on their own and in the presence of unlabeled haemocytes. Both miracidia (A and B) and sporocysts (C and D) transformed *in vitro* for 96 hours fail to demonstrate autofluorescence. Sporocysts transformed for 30 hours and exposed to M-line haemocytes that were not fluorescently labelled (E and F) also fail to produce a fluorescent signal.
(TIF)

S8 Fig Analysis of SmLeish in infected M-line *B. glabrata* and *S. mansoni* ES products. Using the anti-SmLeish antibody in an ELISA, SmLeish was measured in infected M-line *B*. *glabrata*. Cell-free plasma from five snails was assessed in triplicate and compared with three non-challenged control snails as well as ES products. The ELISA was calibrated using a serial dilution of rSmLeish, and the 0.25μg/mL values are shown.
(TIF)
