# Supplementary figures and images for: A metalloprotease produced by larval Schistosoma mansoni facilitates infection establishment and maintenance in the snail host by interfering with immune cell function
Source: PLoS Pathog. 2018 Oct 29;14(10):e1007393. doi: 10.1371/journal.ppat.1007393 (PMC6224180; doi:10.1371/journal.ppat.1007393)

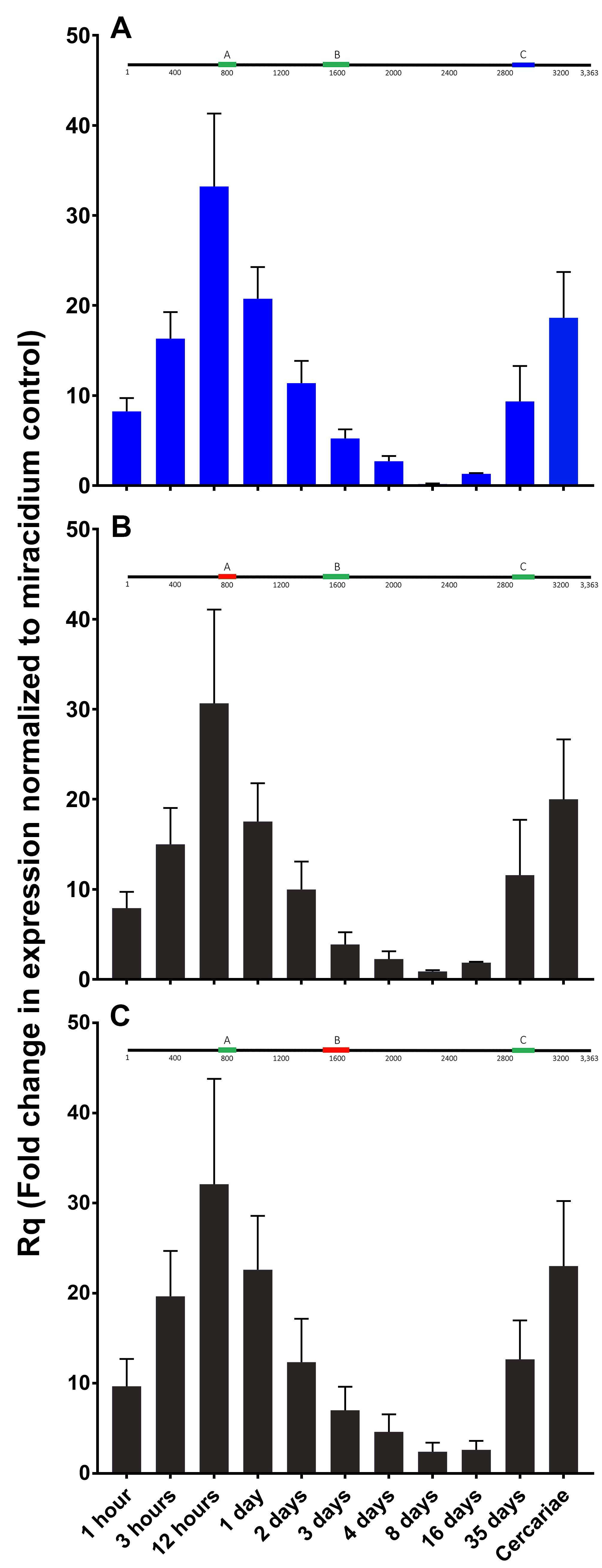

Supplement: S1 Fig — RT-qPCR results do not vary in a statistically significant manner at any time point among three separate sets of primers and probes used to assess the transcript abundance of SmLeish. The primer/probe combination in panel A was used for further analysis such as knock down efficiency, while the primer/probe combinations in panel B and C serve to rule out alternative splicing. (TIF) [file ppat.1007393.s001.tif]

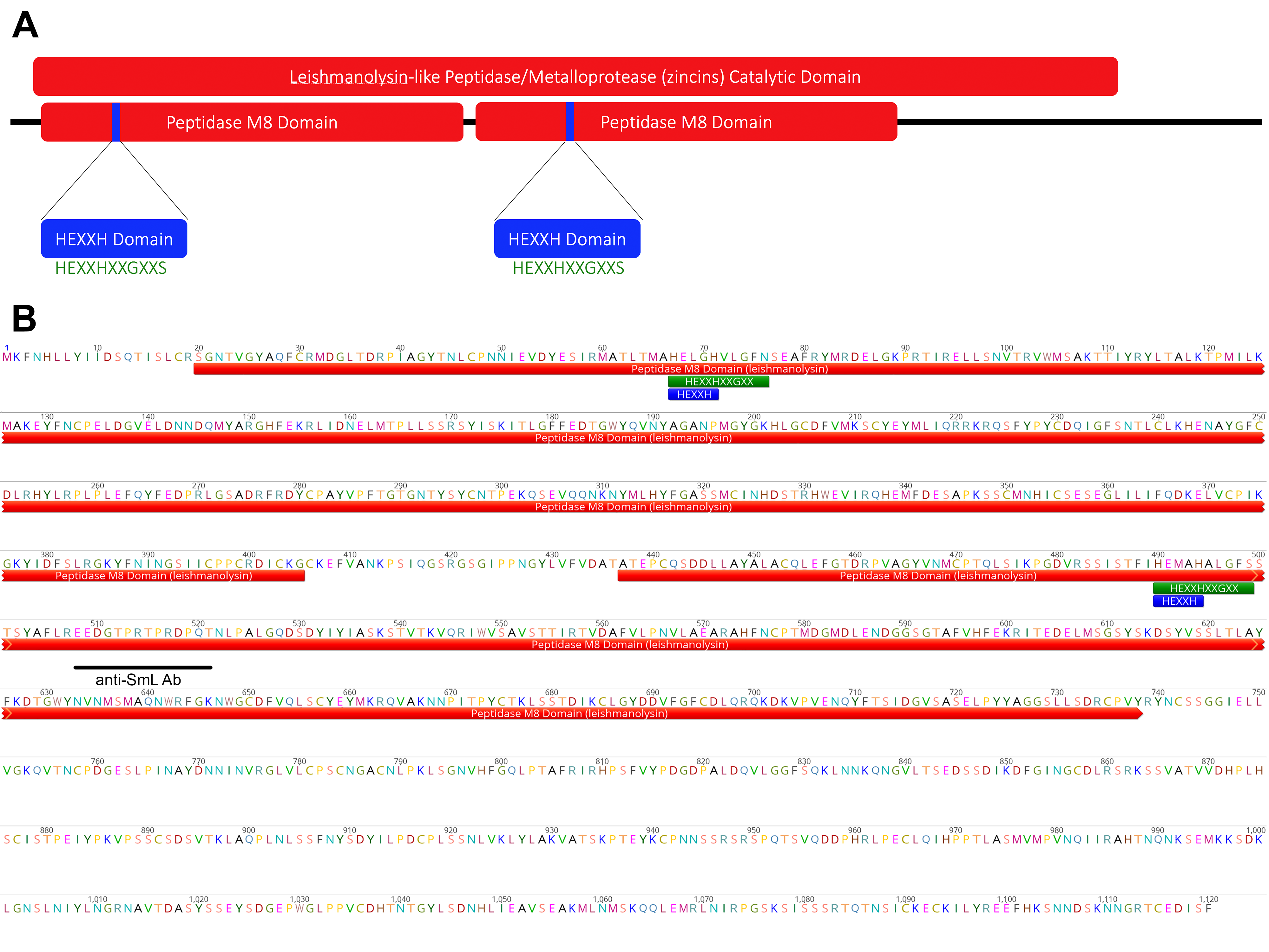

Supplement: S2 Fig — A) SmLeish features a predicted Leishmanolysin-like Peptidase/Metalloprotease (zincins) catalytic domain, which is in turn composed of two predicted Peptidase M8 domains. Within each of these domains resides the canonical HEXXH motif (blue) found in all zinc metalloproteases, which are components of the HEXXHXXGXXS motifs (green) seen across Metzincin Metalloproteases. B) The amino acid sequence of SmLeish highlights the presence of two Peptidase M8 domains and their respective active site motifs. The peptide sequence against which the anti-SmLeish antibody was derived is indicated by the black band. (TIF) [file ppat.1007393.s002.tif]

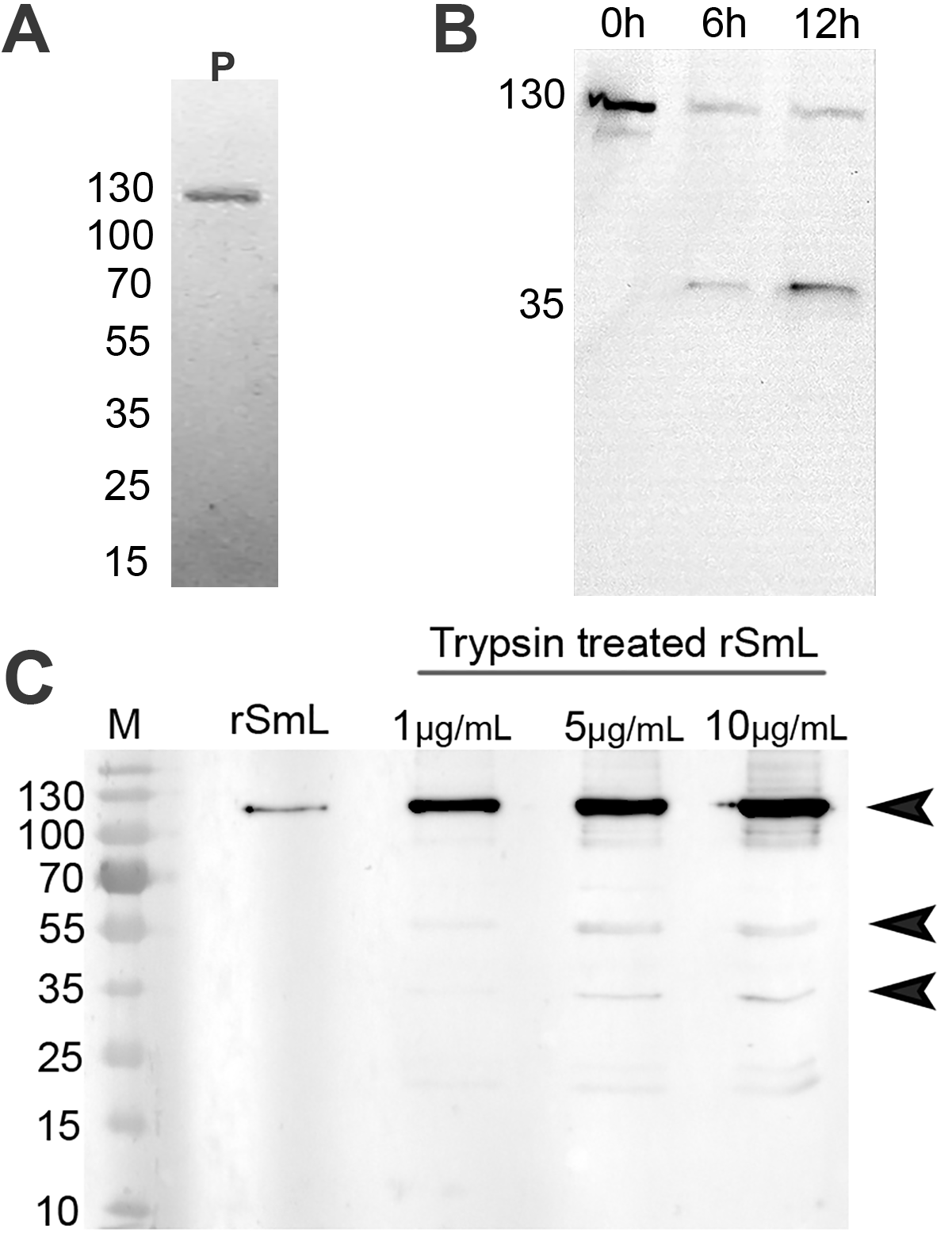

Supplement: S3 Fig — A) SDS-PAGE of the purified rSmLeish demonstrating a pure sample. B) Recombinant SmLeish cleaved using 5ug/ml trypsin for 0, 6, and 12 hours demonstrate the occurrence of a ~48kDa cleavage product, which increases in abundance following longer incubation times. C) Western blot of purified rSmLeish (rSmL) and rSmLeish following a one-hour incubation with 1, 5 and 10μg/mL trypsin at 37C. Cleavage products were detected at ~50kDa and ~35 kDa, as well as at ~25 and 20kDa. MS/MS analysis of the 50 and 35kDa cleavage products indicates that they possess peptide fragments of SmLeish (SmLeish peptide containing products indicated by arrows). (TIF) [file ppat.1007393.s003.tif]

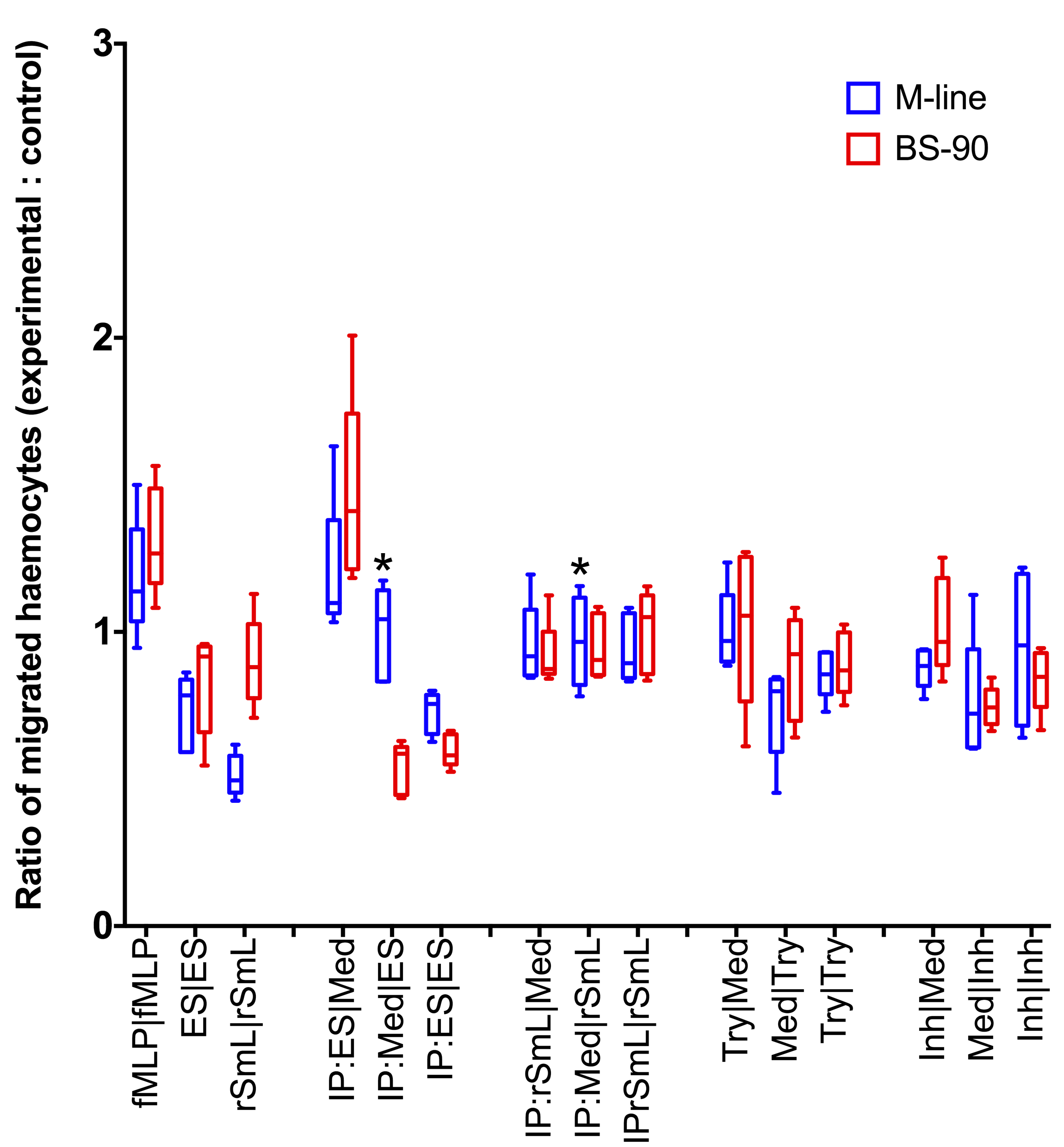

Supplement: S4 Fig — Additional dual well and trypsin/1,10-phenanthroline treatment controls for the haemocyte migration assay. Additionally, immunoprecipitation (IP) of SmLeish from ES products or out of the rSmL treatments provides further evidence that SmLeish is negatively impacting M-line haemocyte motility across the 5μm pore membrane. * indicate significant difference between indicated treatment and the non-SmLeish immunoprecipitated treatment shown in Fig 4A. (TIFF) [file ppat.1007393.s004.tiff]

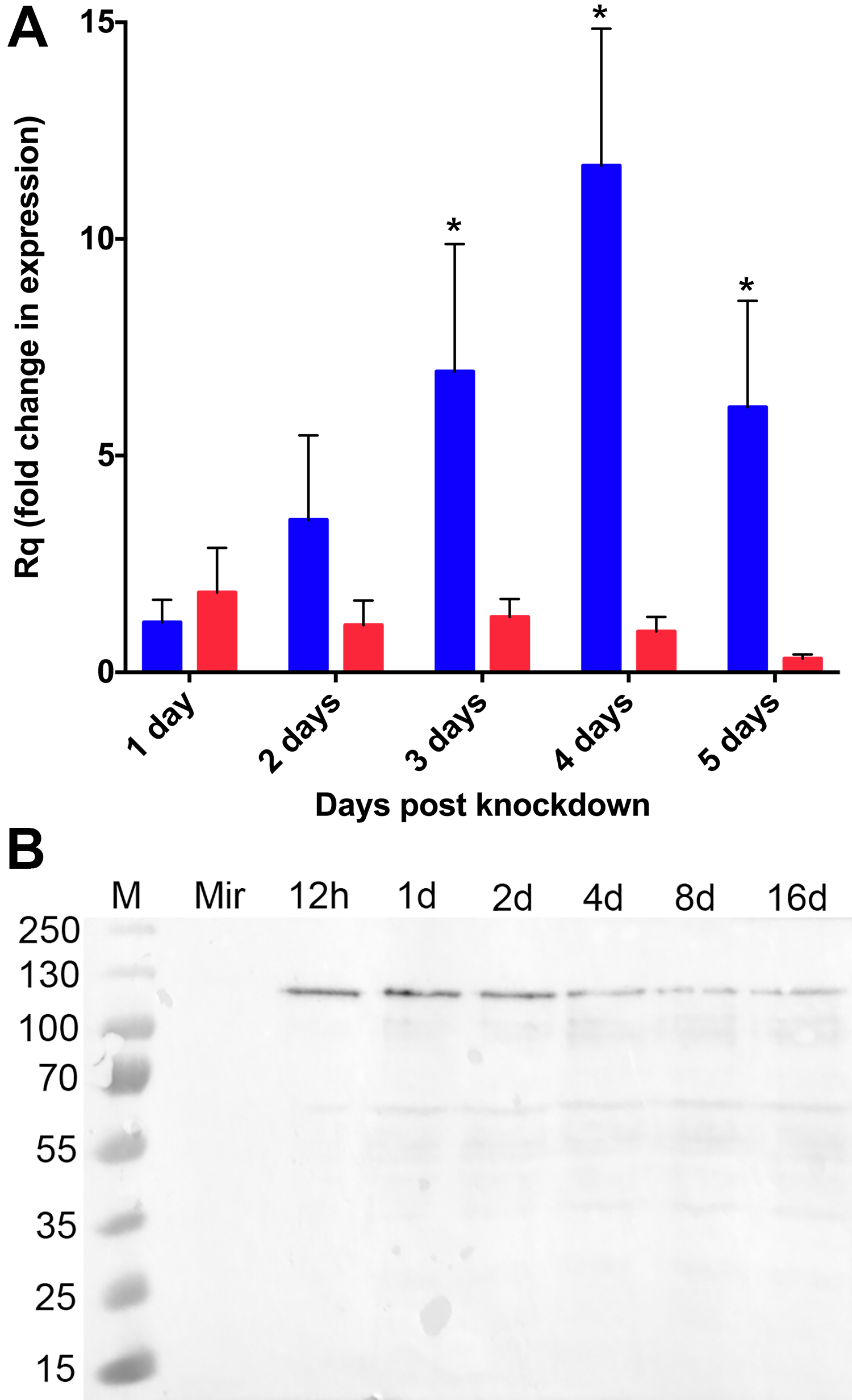

Supplement: S5 Fig — Assessment of in vitro transformed S. mansoni sporocyst expression of SmLeish following SmLeish knockdown (red bars) was compared to GFP knockdown controls (blue bars). Knockdown was significant at the transcriptional level as early as 2 days post incubation and prevented increases in transcript abundance that are traditionally observed for SmLeish by 3 days post transformation (A). Western blot analysis confirmed protein-level knockdown occurs as well, but is less evident for the larger SmLeish protein, while the ~48kDa soluble form of SmLeish is not detectable following knockdown (B). (TIF) [file ppat.1007393.s005.tif]

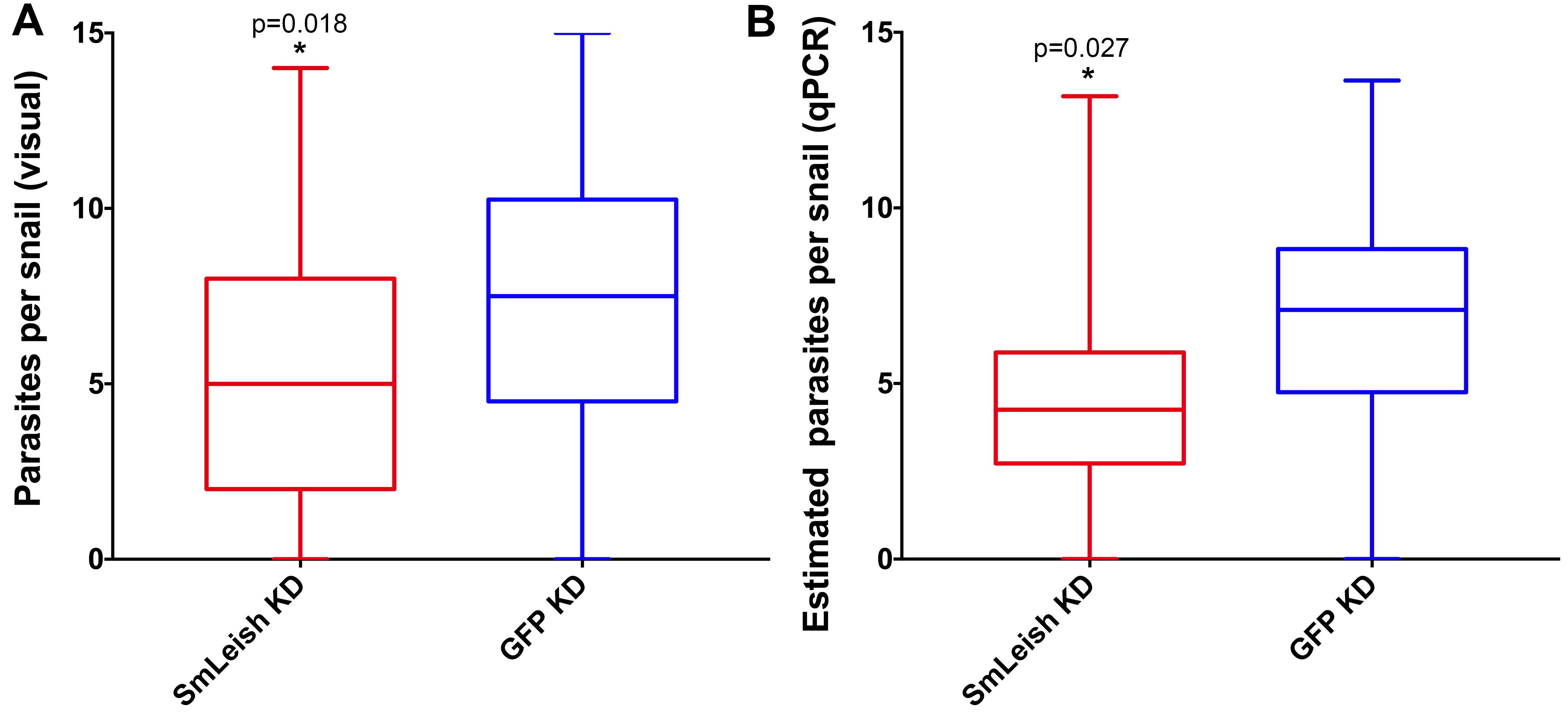

Supplement: S6 Fig — Labeling of the S. mansoni miracidia using a membrane fluorescent dye allowed us to visualize the larval parasites within the head-foot of challenged M-line B. glabrata. This approach enabled us to count the number of sporocysts within the head-foot at 4 days post challenge using 15 labelled miracidia. A) Fewer SmLeish knockdown parasites were observed via visual examination within the head-foot on average compared to GFP knockdown controls. B) qPCR results also suggested a decrease in the among of SmLeish knockdown parasites present within the snail when compared to GFP knockdown controls. (TIF) [file ppat.1007393.s006.tif]

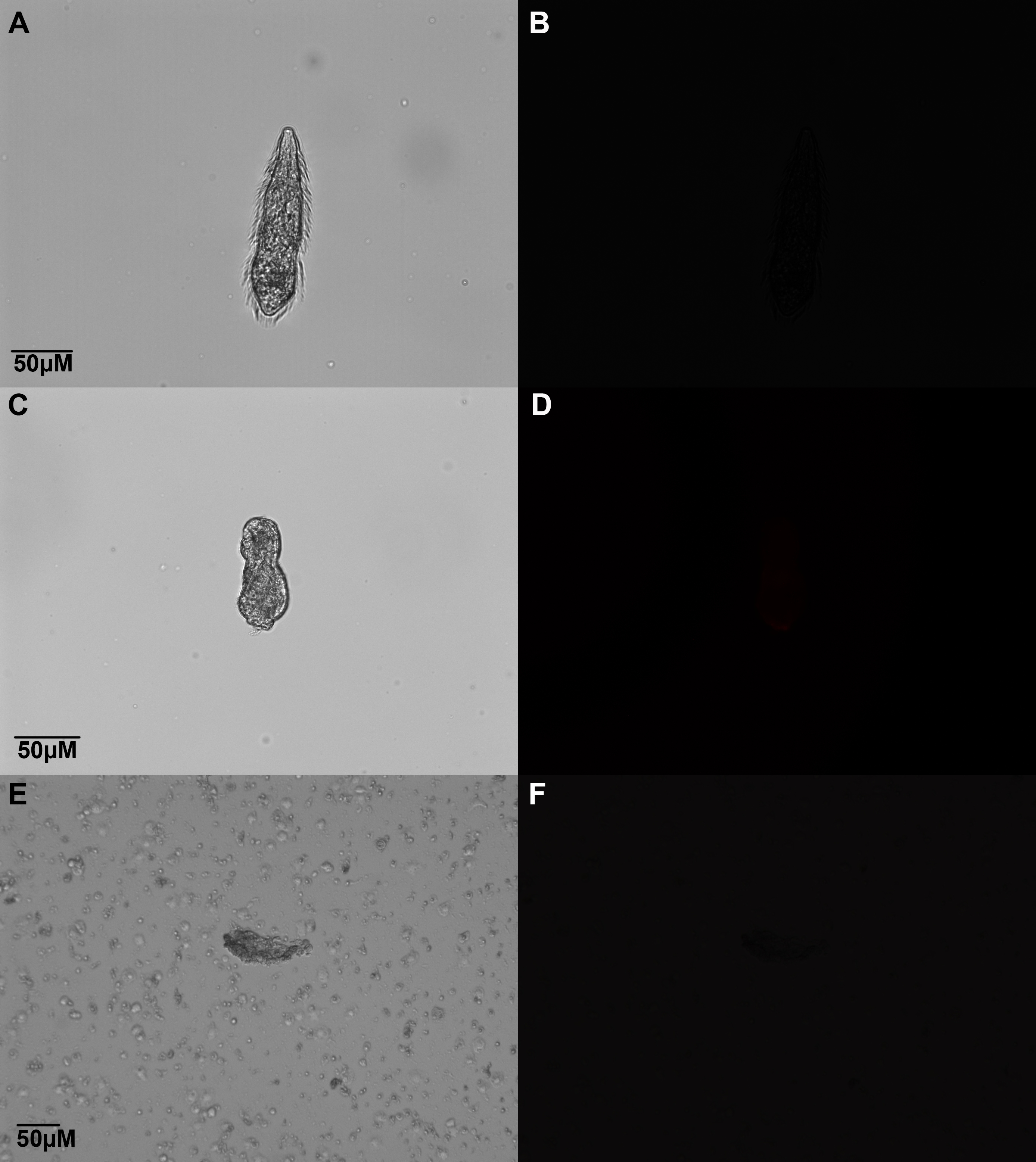

Supplement: S7 Fig — S. mansoni larvae fail to autofluorescence on their own and in the presence of unlabeled haemocytes. Both miracidia (A and B) and sporocysts (C and D) transformed in vitro for 96 hours fail to demonstrate autofluorescence. Sporocysts transformed for 30 hours and exposed to M-line haemocytes that were not fluorescently labelled (E and F) also fail to produce a fluorescent signal. (TIF) [file ppat.1007393.s007.tif]

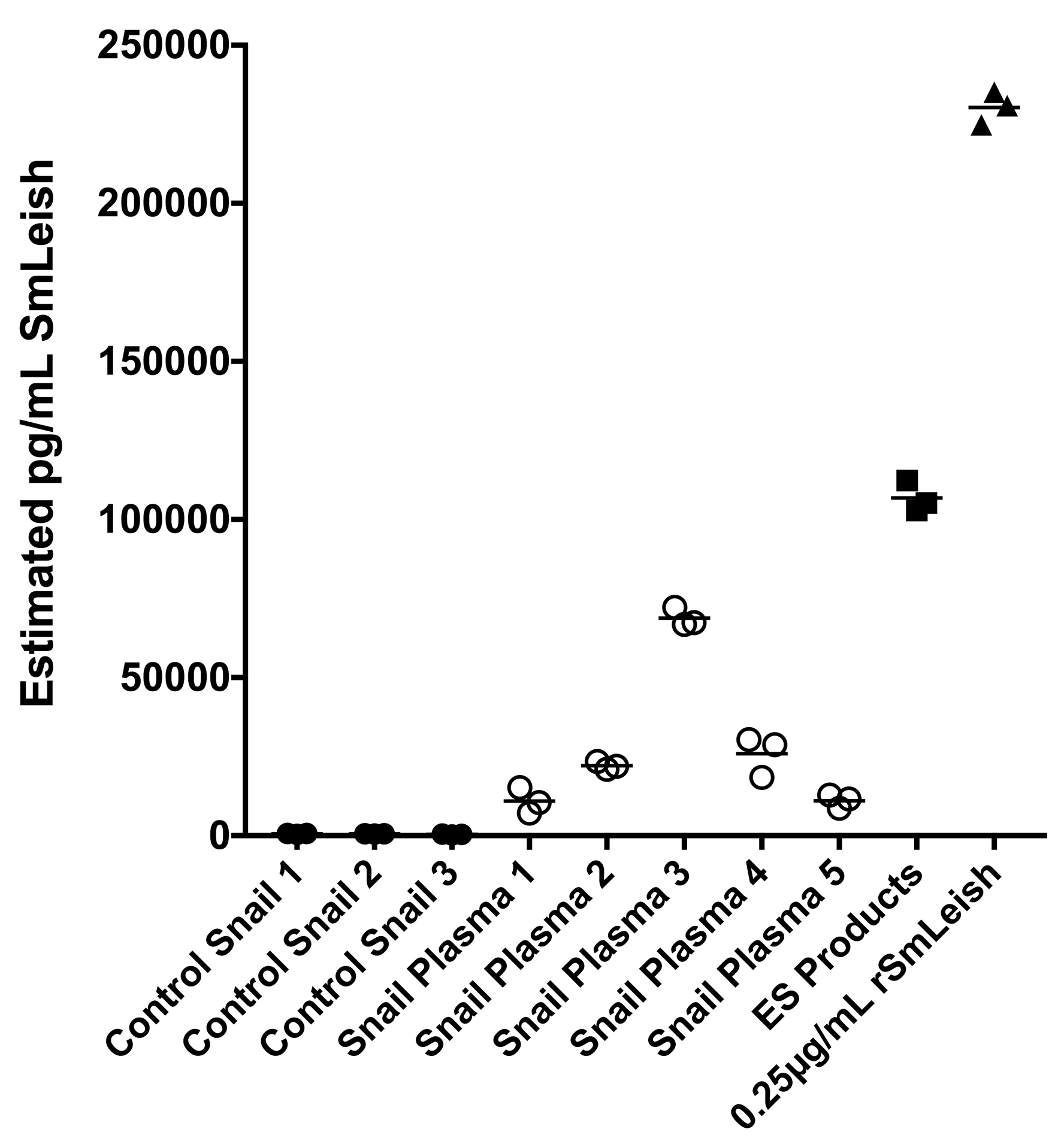

Supplement: S8 Fig — Using the anti-SmLeish antibody in an ELISA, SmLeish was measured in infected M-line B. glabrata. Cell-free plasma from five snails was assessed in triplicate and compared with three non-challenged control snails as well as ES products. The ELISA was calibrated using a serial dilution of rSmLeish, and the 0.25μg/mL values are shown. (TIF) [file ppat.1007393.s008.tif]
